# Automatic coronary artery segmentation of CCTA images using UNet with a local contextual transformer

**DOI:** 10.3389/fphys.2023.1138257

**Published:** 2023-08-22

**Authors:** Qianjin Wang, Lisheng Xu, Lu Wang, Xiaofan Yang, Yu Sun, Benqiang Yang, Stephen E. Greenwald

**Affiliations:** ^1^ School of Computer Science and Engineering, Northeastern University, Shenyang, China; ^2^ College of Medicine and Biological and Information Engineering, Northeastern University, Shenyang, China; ^3^ Department of Radiology, General Hospital of Northern Theater Command, Shenyang, China; ^4^ Key Laboratory of Cardiovascular Imaging and Research of Liaoning Province, Shenyang, China; ^5^ Blizard Institute, Barts and the London School of Medicine and Dentistry, Queen Mary University of London, London, United Kingdom

**Keywords:** coronary artery segmentation, 3D-Unet, local contextual transformer, dense residual connection, convolutional neural network

## Abstract

Coronary artery segmentation is an essential procedure in the computer-aided diagnosis of coronary artery disease. It aims to identify and segment the regions of interest in the coronary circulation for further processing and diagnosis. Currently, automatic segmentation of coronary arteries is often unreliable because of their small size and poor distribution of contrast medium, as well as the problems that lead to over-segmentation or omission. To improve the performance of convolutional-neural-network (CNN) based coronary artery segmentation, we propose a novel automatic method, DR-LCT-UNet, with two innovative components: the Dense Residual (DR) module and the Local Contextual Transformer (LCT) module. The DR module aims to preserve unobtrusive features through dense residual connections, while the LCT module is an improved Transformer that focuses on local contextual information, so that coronary artery-related information can be better exploited. The LCT and DR modules are effectively integrated into the skip connections and encoder-decoder of the 3D segmentation network, respectively. Experiments on our CorArtTS2020 dataset show that the dice similarity coefficient (DSC), Recall, and Precision of the proposed method reached 85.8%, 86.3% and 85.8%, respectively, outperforming 3D-UNet (taken as the reference among the 6 other chosen comparison methods), by 2.1%, 1.9%, and 2.1%.

## 1 Introduction

Cardiovascular disease is a major cause of death worldwide and its most common manifestation is coronary artery disease (CAD) (Jayaraj et al., 2019). Early diagnosis of CAD, especially coronary artery stenosis and atherosclerosis, is essential for subsequent treatment. As a non-invasive screening method, Computed Tomography Angiography (CTA) has been widely used for this purpose (Raff, 2007). However, coronary CTA (CCTA) images have the typical shortcomings of medical images, such as unbalanced foreground-background distribution, small targets, and unstable image quality (Kroft et al., 2007). This instability results from differences in scanning equipment and variations in patient motion during scanning, which affect the consistency of image quality. Radiologists can manually assess the site of stenosis and plaque in coronary arteries, but this is not only time-consuming but also prone to misdiagnosis and omission (Ghekiere et al., 2017). Furthermore, clinical workforce resources are limited, so there is a drive to employ computers to help physicians analyze coronary artery images. Segmentation of coronary arteries in these images is a prerequisite for automating the diagnosis and analysis these tissues. (Mihalef et al., 2011; Tesche et al., 2018). Given the current difficulties in automated diagnosis of CCTA images, there is a need to develop more effective methods for segmenting the coronary arteries contained therein.

In previous research, traditional methods have achieved some notable success in the field of vessel segmentation. These methods include techniques based on image processing, morphological operations, and traditional machine learning algorithms. More than 10 years ago, Lesage et al. (2009) provided further insights into vessel segmentation approaches, which do not involve deep learning. These include the use of region-based methods, edge detection, and active contour models, among others. Orujov et al. (2020) proposed a contour detection algorithm for retinal blood vessels using Mamdani (Type-2) fuzzy rules; the method enhanced contrast with contrast-limited adaptive histogram equalization, removed noise using a median filter, calculated image gradients, and classified pixels as edges based on fuzzy rules considering gradient magnitude and direction, ultimately obtaining segmentation of the blood vessels. Yang et al. (2020) proposed an improved multi-scale enhancement method based on Frangi filtering to enhance the contrast between vessels and other objects in the image, and used an improved level set model to segment vessels from both the enhanced and original grayscale images. Cheng et al. (2015) applied thresholding and morphological operations to preprocessed images, obtaining an initial outline of blood vessels, which were then segmented using an active contour framework based on a B-snake model with a constraint force to prevent leakage into adjacent structures. Kerkeni et al. (2016) proposed a multiscale region growing (MSRG) technique for segmenting coronary arteries in 2D X-ray angiography images, beginning with image enhancement using a multi-scale vascularity filter and a contrast enhancement technique, followed by identifying initial seed points by thresholding and manually selecting points with a high density of vascularity, and finally employing an iterative region growing approach to obtain the segmentation. Although these methods have to some extent helped address vessel segmentation tasks, they still have shortcomings, such as sensitivity to noise, dependency on manual intervention, and difficulty in handling complex vessel structures or poor contrast images.

Recently, deep learning methods have performed extremely well in the segmentation of medical images and have been shown to significantly outperform traditional methods in accuracy. Artificial intelligence has also found extensive applications in cardiothoracic fields, particularly in diagnostic imaging (Sharma et al., 2020). UNet (Ronneberger et al., 2015) is a classical network in the field of biomedical image segmentation and has become a benchmark in this domain (Liu et al., 2020). The network has a U-shaped structure consisting of an encoder, a decoder, and skip connections, which allow it to acquire both spatial and semantic information simultaneously. 3D-UNet (Çiçek et al., 2016), as an extension of UNet, is used for 3D image segmentation. The input is a volume instead of a slice so that interslice information can be exploited, and the convolution operation is changed from 2D to 3D accordingly. A typical 3D-UNet consists of four stages for both the encoder and the decoder. VNet (Milletari et al., 2016), which has also been proposed for processing 3D medical images, is similar to 3D-UNet in terms of network structure. The differences are that it uses convolution operations instead of pooling operations for upsampling and downsampling, and it also introduces residual connections in both the encoder and decoder.

Due to its excellent performance, many studies have employed 3D-UNet as a baseline network and improved upon it. As to the encoding and decoding path, some variants of 3D-UNet add residual connections to the convolution and deconvolution operations in the encoding and decoding stages (Lee et al., 2017; Qamar et al., 2020). Furthermore, some variants of 3D-UNet introduce dense connections between the fine and coarse feature maps to improve the transfer of feature information (Li et al., 2018; Bui et al., 2019; Zhang Y et al., 2020; Pan et al., 2021). Song et al. (2022) incorporated dense blocks into the encoder for effective feature extraction and applied residual blocks to the decoder for feature rectification. Several works have introduced attention mechanisms into UNet (Islam et al., 2020; Jin et al., 2020; Li et al., 2021). For example, channel attention (Li et al., 2021) and spatial attention (Islam et al., 2020) have been added to the decoder. Spatial attention focuses more on the target region, while channel attention estimates the importance of individual features. However, accurate segmentation of medical images requires rich contextual information to resolve ambiguities, and these methods do not make effective use of such information.

The Transformer model (Vaswani et al., 2017) proposed in the Natural Language Processing field has fundamentally changed the way that machines work with text data. Inspired by this, many recent studies have adapted the Transformer model for computer vision applications. For instance, Vision Transformer (Dosovitskiy et al., 2021) divides images into fixed-size patches, and these patches are regarded as words and fed into the Transformer for image recognition. Related works that utilize the Transformer for medical image segmentation have also performed well. VT-UNet (Peiris et al., 2022) uses window-based Transformers as encoders and decoders to construct a U-shaped network for 3D Tumor Segmentation. UNETR (Hatamizadeh et al., 2022) applies the original Transformers as encoders in a U-shaped network to learn the input representation and capture global multi-scale information, while the decoders remain as traditional convolutional modules. UCTransNet (Wang et al., 2022a) introduces the channel Transformer to replace the skip connection of U-Net for more effective encoder-decoder feature connection and hence more accurate segmentation of medical images. AFTer-UNet (Yan et al., 2022) replaces the convolution with a Transformer in the last layer of the UNet. MT-UNet (Wang et al., 2022b) proposes a mixed Transformer and embeds it into the deeper layers of UNet. The mixed Transformer first calculates self-affinities using an efficient local-global self-attention mechanism and then exploits the relations between data samples with an external attention.

Besides the improvements in UNet and the introduction of the attention mechanism, several other network architectures have been employed for coronary artery segmentation. Lei Y et al. (2020) introduced an improved 3D attention into a fully convolution network (FCN) to automatically segment the coronary arteries in CCTA images. Tian et al. (2021) used VNet for initial segmentation and then used region growing to further segment the image, thus obtaining complete and smooth-edged coronary arteries. Gao et al. (2021) conducted coronary centerline extraction and lumen segmentation jointly on CCTA images to address the breakage issue, employing a Graph Convolutional Network (GCN) for the segmentation of the coronary lumen. Some studies use specific features of coronary vessels for segmentation. For instance, Kong et al. (2020) focused more on the anatomical structure of coronary arteries and proposed incorporating a tree-structured convolutional gated recurrent unit into the fully convolutional neural network. Ma et al. (2020) are more concerned with the continuity of the vessels and used a novel region growing method to segment coronary arteries, which considers a variable sector search area within each region. Wolterink et al. (2019) focused more on tubular surfaces and employed graph convolutional networks to forecast the spatial coordinates of vertices within a tubular surface mesh, thus segmenting the lumen of the coronary artery. Other studies have adopted a two-stage framework to achieve coronary artery segmentation in CCTA images. For instance, in the first stage, cardiac segmentation is performed, followed by slicing the cardiac region and segmenting the coronary arteries within the local sliced region. This procedure can alleviate the foreground-background imbalance problem (Dong et al., 2022). Wang et al. (2022c) adopted a similar approach in which the first stage involves a rough segmentation of the 3D image and in the second, the segmentation network is fed with the original 2D images and the 2D images resulting from slicing the 3D segmentation.

However, these existing methods still have shortcomings and we aim at tackling some of them in this work. First, as a proportion of the coronary arteries are of small diameter and thus appear as very thin lines in images, simply increasing the number of convolutional layers in the encoding or decoding blocks of UNet would not improve the segmentation accuracy, because the information in the shallow-layer features, which is necessary for segmenting details, may be lost when the convolution operation goes deeper. Although the traditional residual module (He et al., 2016) can complement the shallow-layer information, it is not sufficient for coronary artery segmentation (as demonstrated in Table 4 in the results and discussion section). Existing research has explored the idea of combining residual learning and dense connections to enhance feature extraction and fusion capabilities in 2D image recognition tasks (Zhang Z et al., 2020; Zhang et al., 2021). However, these approaches are tailored for specific tasks and datasets, and directly applying them to 3D-UNet could result in a large network due to its dense concatenation. Therefore, there is a need to adapt this concept and we have consequently proposed a module specifically designed for 3D coronary artery image segmentation. Second, with similar Hounsfield Unit (HU) values, the feature representations of coronary arteries in the inner layers of a CNN network are likely to be similar to those of other blood vessels such as veins and the ascending aorta. To deal with this, the attention mechanism can be used to enhance the weighting of the coronary regions. However, traditional self-attention computes an attention matrix based on isolated query-key pairs, which may focus more on segmenting the main part of coronary artery and ignore the ends and regions with low concentrations of contrast medium. There has been research on transformers that focus on local context information (Li et al., 2022), and this has been used for 2D image recognition. However, this approach does not simultaneously extract local context information for Q and V, which may limit its feature representation ability. There is a need for a module suitable for 3D-UNet networks for segmentation tasks and to improve the attention mechanism to better capture the local context information of Q and V. This will enhance the feature representation ability of the network.

Therefore, we aim to extract and fuse a greater number of deep and shallow features than the residual module. To this end, we propose the Dense Residual (DR) module, which is continuously supplemented with preceding convolution features during the convolution process, thus improving the encoding and decoding block of UNet. Then, aiming to concentrate more on the local characteristics of the coronary arteries and reduce the noise information from other organs, we propose the Local Contextual Transformer (LCT) module, which focuses more on the local contextual information by obtaining an attention matrix based on query and contextual-information-enhanced key pairs. In particular, we apply the LCT module after each encoding block to provide more informative features to the decoding block, instead of simply using the skip connection, so that the decoding procedure can focus more on the region’s neighboring the coronary arteries. Using these modules, we have conducted extensive experiments on our CorArtTS2020 dataset and compared the results to the most widely used image segmentation method 3D-UNet and six other segmentation networks commonly used in coronary artery segmentation studies. The code of the proposed method is available at https://github.com/qianjinmingliang/Coronary-Artery-segmentation-with-LCTUnet.

## 2 Materials and methods

### 2.1 Dataset

The dataset used for the experiment (CorArtTS 2020) was provided by the General Hospital of the Northern Theater Command in China. It is a modified version of the one used in our previous work (Song A. et al.) and was acquired using a Philips iCT 256 Scanner, running a 120 kVp protocol. Each slice had a width and height of 512 pixels, and the interval between adjacent slices was 0.45 mm. Each case consisted of between 310 and 390 slices. As shown in Table 1, the CorArtTS2020 dataset consists of 81 cases, of which the numbers of normal subjects and patients were 40 and 41, respectively. The data were randomly divided into training, validation, and test sets in the ratio of 6:1:3, respectively.

**TABLE 1:** The CorArtTS2020 dataset.

	Training set	Validation set	Testing set
Normal subjects	24 cases	4 cases	12 cases
Patients	25 cases	4 cases	12 cases

**FIGURE 1:**
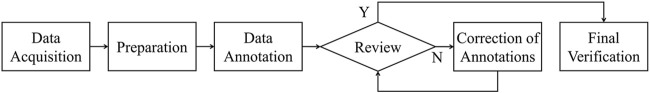
Outline of the annotation process.

The annotation process, summarized in Figure 1, was performed by three experienced radiologists from the same hospital. Initially, data were acquired from the radiology department of the hospital, and the radiologists underwent training to familiarize themselves with the anatomical features and distribution of coronary arteries in CCTA images (Data Acquisition and Preparation). Utilizing specialized medical image processing software, Mimics (Materialise), they carefully annotated the visible contours and branches of the right coronary artery, the left coronary artery, and their branches using the coronal, sagittal, and axial planes (Data Annotation). Upon completion of the annotation process, another experienced cardiovascular imaging radiologist from the same hospital reviewed the annotations (Review). If inaccuracies were found, they were corrected under the guidance of the reviewing radiologist (Correction of Annotations). This iterative and collaborative review process helped to ensure the accuracy of the final labels. Finally, a last check was made to confirm the correctness of all annotations (Final Verification).

**FIGURE 2:**
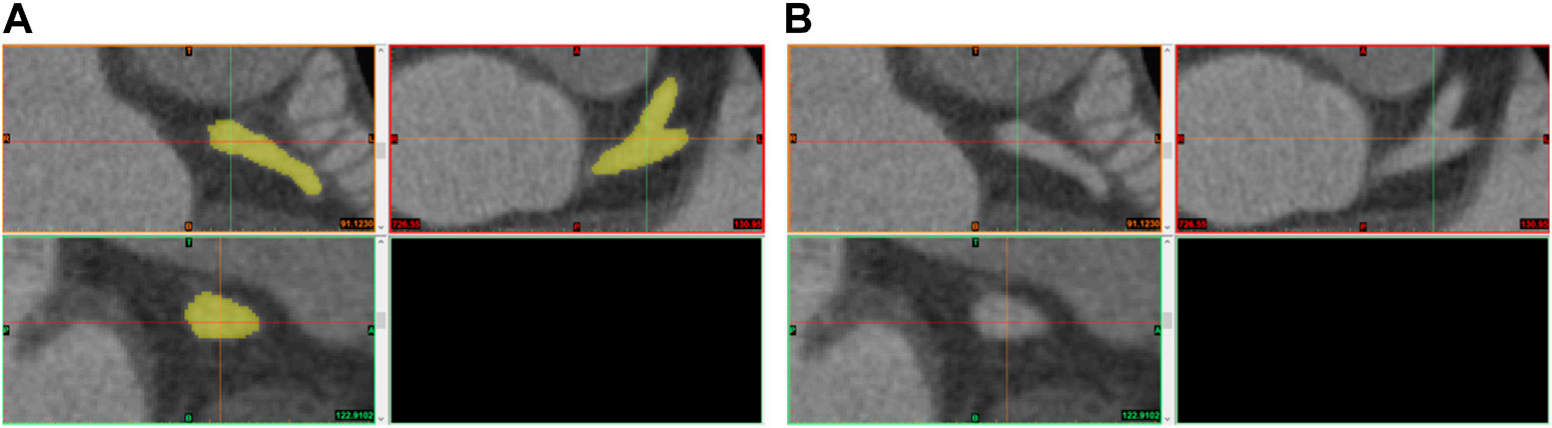
Illustration of the annotation process **(A)** and original CCTA images **(B)**.

Figure 2A provides an example of the annotation process conducted on the Mimics medical image processing software interface, with the annotated regions of the coronary arteries highlighted in yellow. Correspondingly, Figure 2B displays the original CCTA images prior to the annotation process.

**FIGURE 3:**
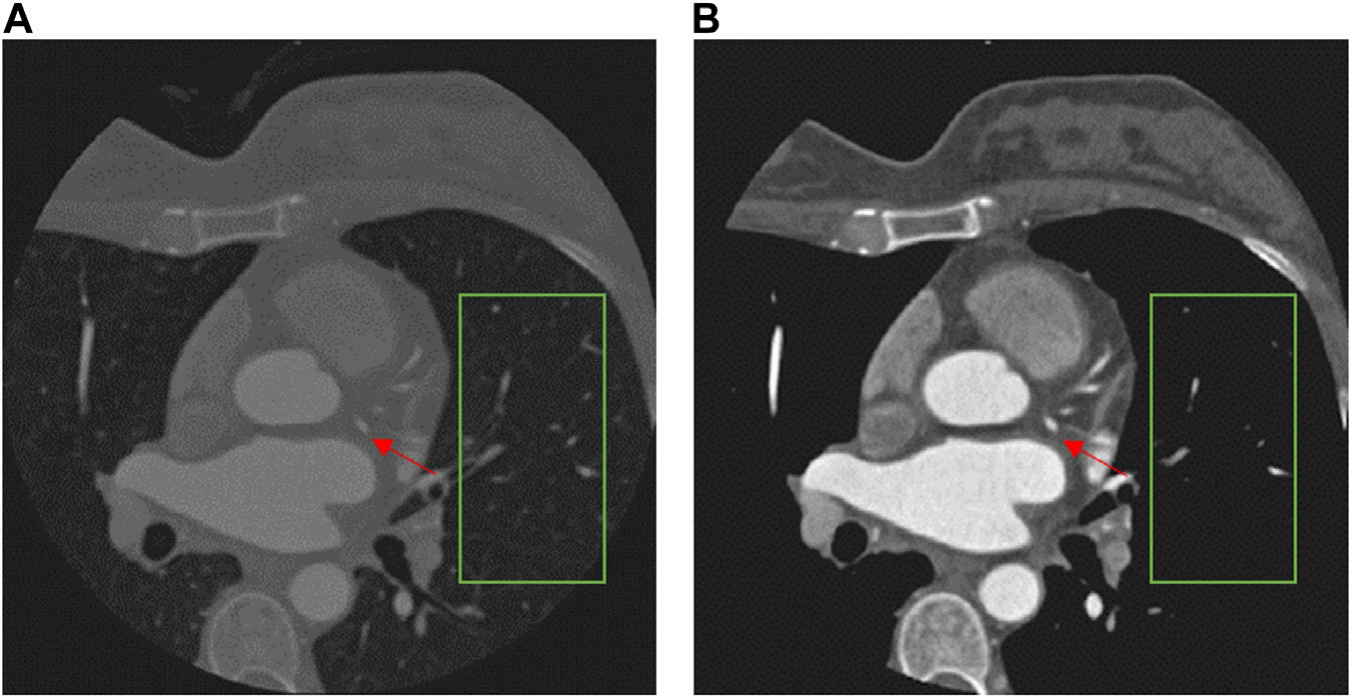
Comparison of a CCTA image before **(A)** and after pre-processing **(B)**.

The CCTA data require pre-processing before being fed into the segmentation network, because different tissues have different radio-densities, giving rise to a wide range of HU values. Highlighting the coronary arteries can improve the segmentation result. However, there is no clear definition of the exact range of HU values for coronary arteries (Marquering et al., 2005; Liu et al., 2013). For our dataset, we therefore conservatively limit the range of HU values in the CCTA data to be within the interval [−260,760] HU, under the guidance of the physicians, and we note that it may not be generalizable to other medical imaging modalities. The result of the data pre-processing is shown in Figure 3. It is notable that the pre-processing effectively removes irrelevant tissues and some noise, as shown in the green box, while making the coronary arteries (red arrows) more distinct. To ensure fair comparison we also used the pre-processed data for all the other comparison methods (Milletari et al., 2016; Çiçek et al., 2016; Lee et al., 2017; Li et al., 2018; Islam et al., 2020; Wang et al., 2022a; Hatamizadeh et al., 2022).

### 2.2 Structure of the DR-lct-unet

**FIGURE 4:**
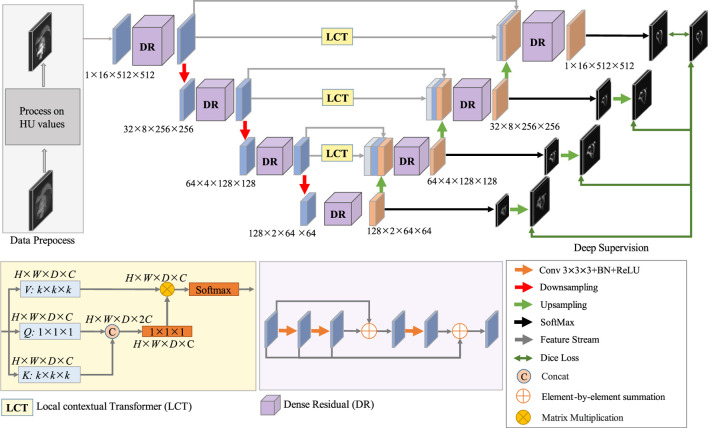
Schematic of the architecture of the proposed DR-LCT-UNet network.

The proposed network structure for coronary artery segmentation is based on the 3D-UNet, to which we have made three modifications. Firstly, the LCT module, which is a novel Transformer-style attention module, is developed to bridge the gap between the features of the encoding and decoding stages before combining them. Secondly, the DR module, which is a mix of residual and dense connections of convolutions, is developed to extract multi-level features for both the encoding and decoding stages. Thirdly, deep supervision is exploited to facilitate the training process of the network. The architecture of the proposed DR-LCT-UNet is shown schematically in Figure 4.

Specifically, in the encoding process, the pre-processed image is fed into the network, and its size is 1 × 16 × 512 × 512, where 1 is the channel size, 16 is the thickness (i.e., the number of slices) of the input volume, and the height and width are 512. There are four layers in the encoding stage, in each of the first three layers, the features are first extracted and then downsampled, while the fourth layer only performs feature extraction. In order to extract rich feature representations for the coronary arteries, the DR module is used in the encoding path, as it is able to extract deeper features while retaining more detailed ones than traditional convolution.

For the decoding process, as the decoding features are quite different from the encoding features after several sampling and convolution operations, the LCT module is used before performing decoding to fill the semantic gap between the features from the encoding and decoding stages of the same resolution level. Consequently, the input to each decoding layer consists of three parts, i.e., the features from the LCT module of the same level, the features from the encoding layers and the features from the previous decoding layers. These features are first concatenated and then decoded by the proposed DR module in each decoding layer.

Finally, we use a deep supervision strategy (Lee et al., 2015) in the training process to prevent the gradient from disappearing in the early stage of training. To be specific, the SoftMax function is applied at the end of each decoding layer to obtain the feature map used for deep supervision. To compute the segmentation loss for deep supervision, each feature map is upsampled to the same size as the input and then the Dice loss is calculated based on the similarity between the feature map and the ground truth.

### 2.3 Structure of the LCT module

In CCTA images, as coronary arteries are smaller compared with nearby structures, and the appearance of coronary arteries and coronary veins is similar, it is necessary to exploit more local contextual information for accurate coronary artery segmentation.

**FIGURE 5:**
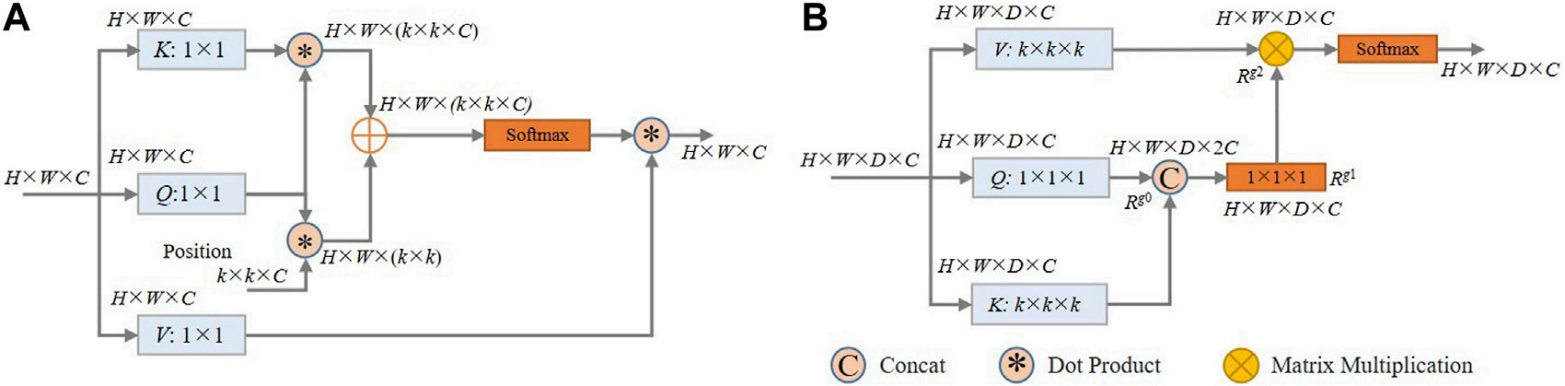
**(A)** Schematic of the architecture of the traditional self-attention model; **(B)** Schematic of the proposed LCT module.

Self-attention (Vaswani et al., 2017) computes an attention matrix based on isolated query-key pairs, as is shown in Figure 5A. *Q*, *K*, and *V* are obtained by 1 × 1 convolution, which only uses the information of each individual location without considering any neighbourhood information. Such operation limits the visual feature representation ability of the resulting embeddings. To deal with this, we propose a local contextual Transformer (LCT) module, the structure of which is shown in Figure 5B).

Specifically, for an input *X* of size *H* × *W* × *D* × *C* (*H*, *W*, *D* and *C* are respectively the height, width, thickness and the number of channels), it is first transformed into queries (*Q*), keys (*K*), and values (*V*) using embedding matrices *W*_*q*_, *W*_*k*_, and *W*_*v*_, respectively. This transformation is represented by *Q* = *XW*_*q*_, *K* = *XW*_*k*_, and *V* = *XW*_*v*_. Instead of using 1 × 1 × 1 convolution to encode each key and value as in traditional self-attention, the LCT module uses *k* × *k* × *k* group convolution over all the neighbouring keys and values within a *k* × *k* × *k* grid to take advantage of local contextual information. That is, the matrices *W*_*k*_ and *W*_*v*_ are set to *k* × *k* × *k* in size, while the matrix *W*_*q*_ is maintained at 1 × 1 × 1 to retain the information of each location in *Q*.

Then, the contextualized keys *K* are concatenated with the queries *Q*. This combined information is then fed into a 1 × 1 × 1 convolution with the ReLU activation function to obtain the attention matrix *R* ^*g*1^∈*R* ^*H*✕*W*✕*D*✕*C*^, thereby learning a feature that integrates local context information with global information.

After that, in a manner similar to traditional self-attention, the values of *V*, which contains the local context information, are multiplied element-by-element with the attention matrix *R* ^*g*1^ to obtain *R* ^*g*2^∈*R* ^*H*✕*W*✕*D*✕*C*^:

$$R^{g2}=R^{g1}⊗V$$ (1)

Finally, a softmax function is applied to *R* ^*g*2^ to yield the output of the LCT module.

In general, the proposed LCT makes use of the local contextual information to enhance the effectiveness of the self-attention calculation, and it can thus adaptively put emphasis on the more relevant regions of the coronary arteries for segmentation. In our implementation, *k* is set to 3 and the optimality of this setting was experimentally validated (see Table 7).

### 2.4 Structure of the DR module

**FIGURE 6:**
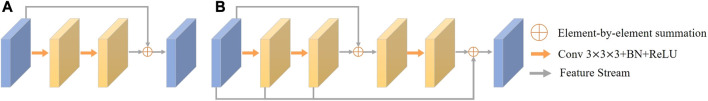
**(A)** Schematic of the architecture of the traditional Residual Module; **(B)** Description of the architecture of the proposed Dense Residual Module.

Traditional residual connection is proposed to solve the degradation problem of deep neural networks. Its structure is shown in Figure 6A, and consists of two consecutive convolution operations and a residual connection. The residual connection is implemented by adding up the features before and after the convolutions. The mathematical description of the original residual connection is

$$Y=X+{Η}^{2}\left(X\right)$$ (2)

where *X* is the input feature, H(*X*) denotes the convolution operation on *X* followed by a ReLU operation, and accordingly, H^*k*^(*X*) denotes *k* successive convolution operations on *X*, each followed by the ReLU operation. Although the residual connection has the effect of preserving the original features, some coronary artery regions in the images are not clear and the corresponding features are not obvious due to the prevalence of narrow areas, such as their distal ends, stenotic regions, and areas with uneven distribution of contrast. Such information may easily get lost during the convolution operation. Therefore, we need to preserve more of the information which might subsequently be lost during the convolution process. To this end, we propose a dense residual (DR) module, as shown in Figure 6B).

Specifically, the DR module has two residual connections which work synergistically to fuse the multi-level features from successive convolution operations. The first residual connection adds the input features to the features obtained after the second convolution operation. After that, one more convolution operation is used to extract additional deeper features. The second residual connection then sums all the previous feature maps, i.e., the input features and the feature maps generated by each of the convolution operations. Thus, the DR module is able to retain features at different convolution levels and extract rich features without information loss. In this way, the features extracted by the DR module can more completely represent the characteristics of coronary arteries. The DR module is defined by the equation:

$$Y=H\left[X+H^{2}\left(X\right)\right]+X+H\left(X\right)+H^{2}\left(X\right)$$ (3)

With the DR module, features in regions with narrow vessels and along low contrast boundaries are enhanced, making more accurate coronary artery segmentation possible.

### 2.5 Loss function

As coronary arteries are of small diameter in comparison with nearby tissues such as the heart, ascending aorta, and the pulmonary artery, the coronary artery segmentation task suffers greatly from the foreground-background imbalance problem. Dice Loss was proposed in 2017 to deal with the imbalance problem in segmentation (Ghekiere et al., 2017). It is well suited to the demands of this study, and has therefore been employed to train our network. The computation of Dice loss is based on the Dice similarity coefficient (DSC), which measures the overlap between two samples, producing results in the range [0,1], i.e., a higher DSC value indicates a higher degree of overlap. The DSC is defined by Eq. 8, and Dice Loss is computed as.

Dice Loss=1−DSC (4)

### 2.6 Deep supervision

For deep supervision, a separate loss is calculated for each decoding layer, which also plays the role of regularization. This strategy, known as deep supervision, leverages the intermediate outputs of the decoding process to guide the training, helping to mitigate the vanishing gradient problem and leading to more discriminative features being learned at all levels. These intermediate losses provide additional guidance to the learning process, which often results in faster convergence.

The loss function used for deep supervision is defined in Eq. 5, where *L*_*k*_ denotes the loss at the decoding layer of depth *k*, and the Dice Loss is defined by Eq. 4. As the output of the first decoding layer has the greatest effect on the performance of the network, we set smaller weights for the losses of the other decoding layers, i.e., *α* < 1. The weight *α* for deep supervision is also gradually decreased during the training process so that at the end of the training the loss reflects the segmentation quality of the last decoding layer.

$$L=L_{1}+\alpha \left(L_{2}+L_{3}+L_{4}\right)$$ (5)

## 3 Experiments and results

### 3.1 Experimental settings

All the experiments were carried out on a GeForce RTX 3090 GPU. The experimental environment was Pytorch 1.7 and the same training process was used for the proposed network and the other compared methods. The input was a volume of size 16 × 512 × 512. The Adam optimizer which uses adaptive moment estimation to speed up convergence was employed to update the network parameters. Due to GPU memory limitations, we chose a batch size of 3 to avoid out-of-memory errors. The parameter settings for the training process are shown in Table 2.

**TABLE 2:** Parameter settings for the training process.

Parameters	Values
Batch size	3
Epochs	180
Learning rate (0< epochs< 100)	10^–5^
Learning rate (100≤ epochs< 160)	10^–6^
Learning rate (160≤ epochs≤ 180)	10^–7^
weight decay factor	5 × 10^−4^
α (0< epochs< 40)	1
α (40≤ epochs< 80)	0.8
α (80≤ epochs< 120)	0.8^2^
α (120≤ epochs< 160)	0.8^3^
α (160≤ epochs≤ 180)	0.8^4^

### 3.2 Evaluation metrics

We applied five commonly used evaluation metrics, i.e., the Dice similarity coefficient (DSC), Recall, Precision, Average Symmetric Surface Distance (ASSD), and Hausdorff Distance (HD), to evaluate the effectiveness of the different methods (Kirişli et al., 2013). DSC describes the similarity between two samples. Recall is the ratio of the number of correctly predicted positive voxels to the actual number of positive voxels. Precision is the proportion of correctly predicted positive voxels to all the voxels predicted to be positive. ASSD describes the average surface distance between two samples. HD describes the maximum distance from a point in the label to a nearest point in the predicted image. The five evaluation metrics were computed according to the following expressions:

$$Recall=\frac{TP}{TP+FN}$$ (6)

$$Precision=\frac{TP}{TP+FP}$$ (7)

$$DSC=\frac{2TP}{2TP+FN+FP}$$ (8)

$$ASSD=\frac{\sum_{S\left(TP+FP\right)}d\left[S\left(TP+FP\right),S\left(TP+FN\right)\right]+\sum_{S\left(TP+FN\right)}d\left[S\left(TP+FN\right),S\left(TP+FP\right)\right]}{\left|S\left(TP+FP\right)\right|+\left|S\left(TP+FN\right)\right|}$$ (9)

$$HD=\max\left(\max_{a\in S\left(TP+FP\right)}\left\{\min_{b\in S\left(TP+FN\right)}\left\|a-b\right\|\right\},\max_{b\in S\left(TP+FN\right)}\left\{\min_{a\in S\left(TP+FP\right)}\left\|b-a\right\|\right\}\right)$$ (10)

where *TP* (True Positives) represents samples correctly identified as coronary arteries; *FN* (False Negatives) denotes samples predicted to be background, but which actually belong to coronary arteries; *FP* (False Positives) indicates samples predicted to be coronary arteries, but which actually belong to the background. *S* (*TP* + *FN*) is the set of actual surface voxels of the coronary arteries, and *S* (*TP* + *FP*) is the set of predicted surface voxels of the coronary arteries. *d* [*sample* ^1^, *sample* ^2^] refers to the shortest distance from *sample* ^1^ to *sample* ^2^. The values of DSC, Recall, and Precision are all in the range of [0,1], and larger values indicate better performance; while for ASSD and HD, smaller values are better.

### 3.3 Experimental results and discussion

#### 3.3.1 Comparison of the different segmentation networks

To assess the quality of the proposed network structure, we have reproduced and retrained some classical and state-of-art methods commonly used for medical image segmentation from scratch. It is noteworthy that our model’s final scores on the test set are not dependent on a single run. Instead, they are computed as the average results from multiple runs, thus enhancing the robustness and stability of our model and preventing the results from being influenced by a specific initialization of the model.

A comparison of the proposed DR-LCT-UNet with the other networks is shown in Tables 3, 4. The proposed network achieves better results than the baseline 3D-UNet in terms of all five evaluation metrics. Specifically, compared with the 3D-UNet, DR-LCT-UNet improves DSC by 2.1%, Recall by 1.9%, Precision by 2.1%, reduces ASSD by 0.188, and reduces HD by 1.861. DR-LCT-UNet also outperforms other networks in terms of DSC, Recall, ASSD, and HD. These results can be seen in Figure 8 which shows the final 3D reconstruction of the segmented coronary arterial tree.

**TABLE 3:** Comparison of segmentation results between various methods (Optimal value for each evaluation metric is shown in bold. In the top row of this and subsequent tables, up arrows indicate that an increase in the evaluation metric implies better performance, the reverse being the case for the down arrows).

Method	DSC↑	Recall↑	Precision↑	ASSD↓	HD↓
3D-UNet (Çiçek et al., 2016)	0.837	0.844	0.837	0.613	30.076
VNet (Milletari et al., 2016)	0.837	0.810	0.872	0.538	33.519
ResUNet (Lee et al., 2017)	0.841	0.832	**0.882**	0.533	29.054
DenseUNet (Li et al., 2018)	0.839	0.826	0.859	0.514	37.413
AttUNet (Islam et al., 2020)	0.843	0.835	0.857	0.506	32.642
UNETR (Hatamizadeh et al., 2022)	0.827	0.784	0.884	0.551	44.071
UCTransNet (Wang et al., 2022a)	0.818	0.821	0.813	1.205	59.128
DR-LCT-UNet (Ours)	**0.858**	**0.863**	0.858	**0.425**	**28.215**

**TABLE 4:** Comparison of the number of parameters and inference time for the different methods.

Method	Parameters (M)	Inference Time (s/case)
3D-UNet (Çiçek et al., 2016)	8.61	17.21
VNet (Milletari et al., 2016)	16.80	18.65
ResUNet (Lee et al., 2017)	9.50	18.50
DenseUNet (Li et al., 2018)	18.10	16.35
AttUNet (Islam et al., 2020)	8.65	17.23
UNETR (Hatamizadeh et al., 2022)	92.58	20.65
UCTransNet (Wang et al., 2022a)	65.60	19.55
DR-LCT-UNet (Ours)	10.70	18.60

The improvements in the various evaluation metrics achieved by the proposed DR-LCT-UNet indicate its superiority in the task of coronary artery segmentation. The improvement of Recall indicates that more coronary arteries are correctly segmented, the improvement of ASSD indicates that the segmentation result differs less from the ground truth, and the improvement of DSC indicates that the overall segmentation is better and closer to the ground truth label. Although the Precision of the networks ResNet, VNet and UNETR, is higher than that achieved by the network proposed here, indicating that they have fewer background voxels mistakenly segmented as coronary arteries, their Recall and DSC scores are much lower than the proposed network, meaning that their segmentation results miss more coronary artery voxels.

#### 3.3.2 Ablation experiments

First, to demonstrate the performance improvement associated with each proposed module of our DR-LCT-UNet, we carried out an ablation study, the results of which are shown in Table 5. It can be seen that, compared with UNet, both the proposed LCT and DR modules consistently improve the five evaluation metrics. Specifically, the LCT module markedly improves the Precision (3.2%), while the DR module substantially improves the Recall (1.9%).

**TABLE 5:** Results of the ablation experiments (Optimal value for each evaluation metric is in bold). Legend: SA: self-attention module; LCT: local contextual Transformer; DR: Dense Residual module; R: Residual block. The ticks indicate which modules are included in each model.

Method	SA	LCT	DR	R	DSC↑	Recall↑	Precision↑	ASSD↓	HD↓
3D-UNet					0.837	0.844	0.837	0.613	30.076
SA-UNet	✓				0.843	0.835	0.857	0.506	32.642
LCT-UNet		✓			0.852	0.846	0.869	0.480	29.057
R-UNet				✓	0.841	0.832	**0.882**	0.533	29.054
DR-UNet			✓		0.852	0.863	0.847	0.494	29.821
DR-LCT-UNet		✓	✓		**0.858**	**0.863**	0.858	**0.425**	**28.215**

The ablation study results demonstrate the effectiveness of the individual LCT and DR modules in enhancing the segmentation performance. The LCT module contributes to a marked improvement in Precision, while the DR module has a considerable impact on Recall. By combining the advantages of both the LCT and DR modules, the DR-LCT-UNet achieves superior performance in terms of DSC, Recall, ASSD, and HD, highlighting the complementary benefits of the two modules.

Second, we have compared the number of parameters and the average inference time for the different modules. As shown in Table 6, the LCT and DR modules only slightly increase the number of parameters and the inference time compared with the SA and Residual modules.

**TABLE 6:** Comparison of the number of parameters and inference time for the different modules.

Baseline	Module	Parameters (M)	Inference Time (s/case)
3D-UNet	—	8.61	17.21
	SA	8.65	17.23
	LCT	8.80	17.23
	R	9.50	18.50
	DR	10.28	18.55
	LCT + DR	10.70	18.60

This result demonstrates that, despite the minor increase in the number of parameters and inference time, the LCT and DR modules achieve much better segmentation accuracy compared to the SA and Residual modules. This demonstrates the effectiveness of the proposed LCT and DR modules in improving segmentation performance without significantly impacting computational complexity.

Third, to support our claim that setting the convolutional kernel *W*_*k*_ and *W*_*v*_ to the same size, i.e., *k* × *k* × *k*, in the LCT module is optimal, we investigated three different strategies for setting the kernel size, the results of which are shown in Table 7. We see that although every kernel size setting strategy improves the segmentation performance compared to that of using the self-attention module (i.e., SA-UNet in Table 5), the first option led to the best performance, i.e., using convolution kernels of the same size for *K* and *V*. In addition, Table 7 shows that using *k* = 3 produces the best segmentation accuracy.

**TABLE 7:** Different structural designs of LCT modules (Optimal values shown in bold).

Q	K	V	DSC↑	Recall↑	Precision↑	ASSD↓	HD↓
1 × 1 × 1	1 × 1 × 1	1 × 1 ×1	0.847	0.842	0.850	0.511	30.015
	3 × 3 ×3	1 × 1 × 1	0.851	0.846	0.867	0.491	29.381
	3 × 3 × 3	3 × 3 × 3	**0.852**	**0.846**	**0.869**	**0.480**	**29.057**
	1 × 1 × 1	3 × 3 × 3	0.849	0.844	0.853	0.509	29.108
	5 × 5 × 5	1 × 1 × 1	0.845	0.840	0.855	0.513	29.277
	5 × 5 × 5	5 × 5 × 5	0.845	0.841	0.854	0.515	29.351
	1 × 1 × 1	5 × 5 × 5	0.844	0.842	0.852	0.520	29.330
	7 × 7 × 7	1 × 1 × 1	0.844	0.843	0.851	0.522	30.164
	7 × 7 × 7	7 × 7 × 7	0.844	0.842	0.849	0.522	30.097
	1 × 1 × 1	7 × 7 × 7	0.843	0.841	0.847	0.525	30.172

This result demonstrates that the sizes of local regions for spatial context extraction of *K* and *V* should be matched. This shows that, contrary to our intuition, obtaining the contextual information from a larger neighbourhood, which will accordingly increase the number of parameters of the LCT module, does not necessarily result in a better segmentation accuracy. It is likely that this is because a larger neighbourhood may introduce more irrelevant information into the segmentation process and thus degrade the segmentation accuracy.

#### 3.3.3 Deep supervision

**FIGURE 7:**
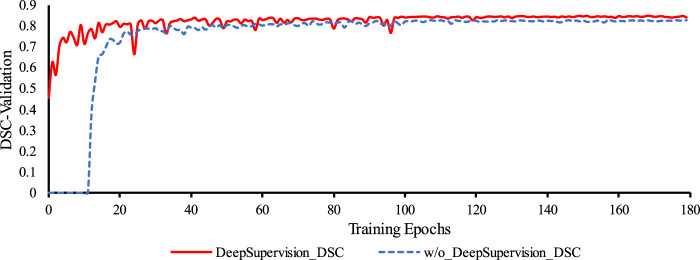
Comparison of the training process of the proposed network with and without Deep Supervision. Legend: w/o: without.

We used deep supervision to prevent gradient disappearance and explosion. As can be seen from Figure 7, with an increase in the number of training epochs, the training is clearly accelerated at the beginning of the process and the DSC value of the validation set also improves. Table 8 confirms this and also shows that deep supervision leads to a slight improvement of the other segmentation metrics.

**TABLE 8:** Results comparison of the proposed method without and with Deep Supervision.

Method	DSC↑	Recall↑	Precision↑	ASSD↓	HD↓
DR-LCT-UNet_w/o_ Deep_Supervision	0.856	0.861	0.855	0.438	28.220
DR-LCT-UNet_ Deep_Supervision	0.858	0.863	0.858	0.425	28.215

The results obtained from incorporating deep supervision demonstrate its benefits in both the network training and the prediction accuracy of the coronary artery segmentation task. By accelerating the training process and enhancing the DSC value of the validation, deep supervision proves to be a valuable technique in optimizing the proposed segmentation network.

#### 3.3.4 Effect of data pre-processing

To show the effectiveness of data pre-processing on the segmentation results, we used the data with and without pre-processing, to train and test the UNet and our DR-LCT-UNet. As shown in Table 9, the segmentation metrics DSC, Recall, Precision ASSD and HD are all clearly improved.

**TABLE 9:** The impact of data pre-processing on the network.

Method	DSC↑	Recall↑	Precision↑	ASSD↓	HD↓
UNet_w/o_Data_preprocess	0.820	0.826	0.820	1.195	60.412
UNet_Data_preprocess	0.837	0.844	0.837	0.613	30.076
DR-LCT-UNet_w/o_Data_preprocess	0.841	0.848	0.840	0.597	29.024
DR-LCT-UNet_Data_preprocess	0.858	0.863	0.858	0.425	28.215

The improvements in the segmentation metrics can be attributed to the fact that truncating the range of HU values can increase the contrast along the boundaries of the coronary arteries, remove some irrelevant tissues from the images and eliminate some noise as well, thus making the network learning and inference more effective.

## 4 Visual illustration of the segmentation results

**FIGURE 8:**
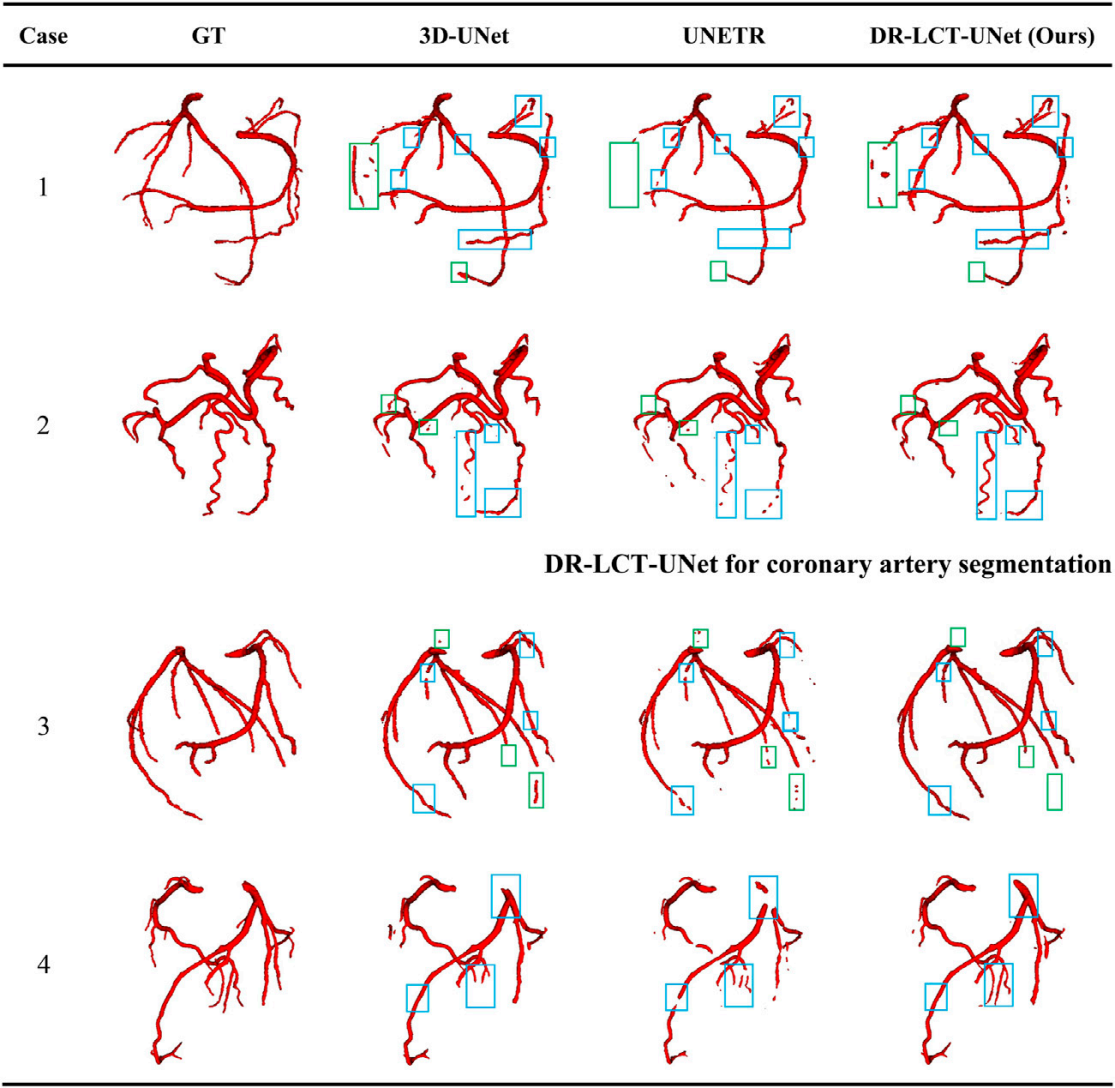
Visualization of the segmentation results from the different methods. Legend: Green boxes: locations where over-segmentation occurs for at least one of the compared methods; blue boxes: locations with under-segmentation.

Figure 8 shows 3D reconstructions of the segmentation results using UNet, UNETR, and the proposed DR-LCT-UNet. Four cases were randomly chosen from the test set, with the first two from normal subjects and the latter two belonging to patients with cardiovascular disease. The segmentation results of our proposed method are better than those of the other two methods, resulting in fewer discontinuities and more complete segmentation at the ends of the coronary arteries.

This performance improvement can be attributed to the Dense Residual (DR) and Local Contextual Transformer (LCT) modules in our model. Specifically, the DR module, through its feature preservation capability at various convolution levels, is key to this enhancement. This module supplements shallow information layers, such as spatial structures and gray-scale features, thereby improving the network’s sensitivity. This enhanced sensitivity facilitates the segmentation of a greater number of coronary arteries. Furthermore, the DR module excels in extracting deeper-level features without compromising the retention of these shallow features, contributing to a more comprehensive feature map for segmentation tasks. On the other hand, the LCT module, serving as an attention mechanism, focuses predominantly on the vicinity of the coronary arteries. It effectively distinguishes these arteries from other vessels with similar CT intensities. When implemented post the encoding block, the LCT module enhances the skip connections, thereby improving the model’s feature representation ability. This enhancement leads to the provision of richer, more diversified features for the decoder, optimizing the feature extraction and representation in our deep learning model. Consequently, the combined operation of the DR and LCT modules results in fewer discontinuities and a more complete and precise segmentation at the ends of the coronary arteries.

While our method does produce fewer false positives compared to the UNet, it has shown a tendency for occasional over-segmentation compared to the UNETR, as demonstrated in case 1 (specifically, the area within the green box). In-depth analysis reveals that this is due to the sensitivity of the Dense Residual (DR) module to shallow information, which occasionally results in the misidentification of structures similar to the coronary arteries, such as veins. This over-sensitivity and the resulting over-segmentation suggest areas of improvement. We acknowledge this limitation and plan to refine our model in follow-up studies, to better distinguish between similar structures.

## 5 Conclusion

The proposed method for coronary artery segmentation, DR-LCT-UNet, alleviates the omission and over-segmentation problems of previous methods for several reasons. Firstly, the data preprocessing enhances the contrast at the boundaries of the coronary arteries and reduces some of the noise in the image, hence improving the segmentation to some extent. Secondly, the proposed Transformer-style LCT module can pay more attention to local contextual information, reducing the semantic gap between the encoding and decoding features, significantly improving the segmentation Precision. Furthermore, the proposed DR module for the encoding stage can preserve multi-level features, reducing the loss of shallow-layer information due to the convolution process. As a result, this improves the Recall of the segmentation. Finally, introducing Deep Supervision to the network improves the efficiency of the training process and also has the effect of regularizing feature extraction for the different decoding layers. The final DSC, Recall, and Precision of the proposed method are 85.8%, 86.3%, and 85.8%, respectively, which are 2.1%, 1.9%, and 2.1% better than the corresponding values for 3D-UNet, the most widely used image segmentation method and the baseline based on which our approach has been developed.

## Data Availability

The original contributions presented in the study are included in the article/Supplementary Material, further inquiries can be directed to the corresponding authors.

## Supplementary material

Supplementary Image 1 Segmentation result of the DR-LCT-UNet (ours) method on CCTA data of case 4.

Supplementary Image 2 Segmentation result of the UNETR method on CCTA data of case 4.

Supplementary Image 3 Segmentation result of the 3D-UNet method on CCTA data of case 4.

Supplementary Image 4 Ground Truth segmentation result on CCTA data of case 4.

Supplementary Image 5 Segmentation result of the DR-LCT-UNet (ours) method on CCTA data of case 1.

Supplementary Image 6 Ground Truth segmentation result on CCTA data of case 2.

Supplementary Image 7 Segmentation result of the 3D-UNet method on CCTA data of case 2.

Supplementary Image 8 Segmentation result of the UNETR method on CCTA data of case 2.

Supplementary Image 9 Segmentation result of the DR-LCT-UNet (ours) method on CCTA data of case 2.

Supplementary Image 10 Ground Truth segmentation result on CCTA data of case 3.

Supplementary Image 11 Segmentation result of the 3D-UNet method on CCTA data of case 3.

Supplementary Image 12 Segmentation result of the UNETR method on CCTA data of case 3.

Supplementary Image 13 Segmentation result of the DR-LCT-UNet (ours) method on CCTA data of case 3.

Supplementary Image 14 Ground Truth segmentation result on CCTA data of case 1.

Supplementary Image 15 Segmentation result of the 3D-UNet method on CCTA data of case 1.

Supplementary Image 16 Segmentation result of the UNETR method on CCTA data of case 1.
