# Supplementary material for: Automatic coronary artery segmentation of CCTA images using UNet with a local contextual transformer
Source: Front Physiol. 2023 Aug 22;14:1138257. doi: 10.3389/fphys.2023.1138257 (PMC10478234; doi:10.3389/fphys.2023.1138257)
Supplement: Supplementary file 1 [file Table1.DOCX]

**TABLE 1.** The CorArtTS2020 dataset.

|  | **Training set** | **Validation set** | **Testing set** |
| --- | --- | --- | --- |
| Normal subjects | 24 cases | 4 cases | 12 cases |
| Patients | 25 cases | 4 cases | 12 cases |

**TABLE 2.** Parameter settings for the training process.

| **Parameters** | **Values** |
| --- | --- |
| Batch size | 3 |
| Epochs | 180 |
| Learning rate (0<epochs<100) | 10^-5^ |
| Learning rate (100≤epochs<160) | 10^-6^ |
| Learning rate (160≤epochs≤180) | 10^-7^ |
| weight decay factor | 5×10^-4^ |
| α (0<epochs<40) | 1 |
| α (40≤epochs<80) | 0.8 |
| α (80≤epochs<120) | 0.8^2^ |
| α (120≤epochs<160) | 0.8^3^ |
| α (160≤epochs≤180) | 0.8^4^ |

**TABLE 3.** Comparison of segmentation results between various methods. Optimal value for each evaluation metric is shown in bold.

| **Method** | **DSC**↑ | **Recall**↑ | **Precision**↑ | **ASSD**↓ |
| --- | --- | --- | --- | --- |
| 3D-UNet(Çiçek et al., 2016) | 0.837 | 0.844 | 0.837 | 0.613 |
| VNet(Milletari et al.,2016) | 0.837 | 0.810 | 0.872 | 0.538 |
| ResUNet(Lee et al., 2017) | 0.841 | 0.832 | 0.882 | 0.533 |
| DenseUNet(Li et al., 2018) | 0.839 | 0.826 | 0.859 | 0.514 |
| AttUNet(Islam et al., 2020) | 0.843 | 0.835 | 0.857 | 0.506 |
| UNETR(Hatamizadeh et al., 2022) | 0.827 | 0.784 | 0.884 | 0.551 |
| UCTransNet(Wang et al., 2022) | 0.818 | 0.821 | 0.813 | 1.205 |
| DR-LCT-UNet(Ours) | 0.858 | 0.863 | 0.858 | 0.425 |

**TABLE 4.** Results of the ablation experiments (Optimal value for each evaluation metric is in bold.). Legend: SA: self attention module; LCT: local contextual Transformer; DR: Dense Residual module; R: Residual block. The ticks indicate that the corresponding module is included in the model.

| **Method** | **SA** | **LCT** | **DR** | **R** | **DSC**↑ | **Recall**↑ | **Precision**↑ | **ASSD**↓ |
| --- | --- | --- | --- | --- | --- | --- | --- | --- |
| 3D-UNet |  |  |  |  | 0.837 | 0.844 | 0.837 | 0.613 |
| SA-UNet | 🗸 |  |  |  | 0.841 | 0.835 | 0.857 | 0.516 |
| LCT-UNet |  | 🗸 |  |  | 0.852 | 0.846 | 0.869 | 0.480 |
| R-UNet |  |  |  | 🗸 | 0.840 | 0.832 | **0.882** | 0.533 |
| DR-UNet |  |  | 🗸 |  | 0.852 | 0.863 | 0.847 | 0.494 |
| DR-LCT-UNet |  | 🗸 | 🗸 |  | **0.858** | **0.863** | 0.858 | **0.425** |

**TABLE 5.** Comparison of the number of parameters and inference time for the different modules.

| **Baseline** | **Module** | **Parameters (M)** | **Inference Time (s/case)** |
| --- | --- | --- | --- |
| 3D-UNet | / | 8.61 | 17.21 |
|  | SA | 8.65 | 17.23 |
|  | LCT | 8.80 | 17.23 |
|  | R | 9.50 | 18.50 |
|  | DR | 10.28 | 18.55 |
|  | LCT+DR | 10.70 | 18.60 |

**TABLE 6.** Different structural designs of LCT modules.

| Q | K | V | **DSC**↑ | **Recall**↑ | **Precision**↑ | **ASSD**↓ |
| --- | --- | --- | --- | --- | --- | --- |
| 1🞨1🞨1 | 1🞨1🞨1 | 1🞨1🞨1 | 0.847 | 0.842 | 0.850 | 0.511 |
|  | 3🞨3🞨3 | 1🞨1🞨1 | 0.851 | 0.846 | 0.867 | 0.491 |
|  | 3🞨3🞨3 | 3🞨3🞨3 | **0.852** | **0.846** | **0.869** | **0.480** |
|  | 1🞨1🞨1 | 3🞨3🞨3 | 0.849 | 0.844 | 0.853 | 0.509 |
|  | 5🞨5🞨5 | 1🞨1🞨1 | 0.845 | 0.840 | 0.855 | 0.513 |
|  | 5🞨5🞨5 | 5🞨5🞨5 | 0.845 | 0.841 | 0.854 | 0.515 |
|  | 1🞨1🞨1 | 5🞨5🞨5 | 0.844 | 0.842 | 0.852 | 0.520 |
|  | 7🞨7🞨7 | 1🞨1🞨1 | 0.844 | 0.843 | 0.851 | 0.522 |
|  | 7🞨7🞨7 | 7🞨7🞨7 | 0.844 | 0.842 | 0.849 | 0.522 |
|  | 1🞨1🞨1 | 7🞨7🞨7 | 0.843 | 0.841 | 0.847 | 0.525 |

**TABLE 7.** Results comparison of the proposed method without and with Deep Supervision. Legend: w/o: without.

| **Method** | **DSC**↑ | **Recall**↑ | **Precision**↑ | **ASSD**↓ |
| --- | --- | --- | --- | --- |
| DR-LCT-UNet_w/o_ Deep_Supervision | 0.856 | 0.861 | 0.855 | 0.438 |
| DR-LCT-UNet_ Deep_Supervision | 0.858 | 0.863 | 0.858 | 0.425 |

**TABLE 8.** The impact of data pre-processing on the network.

| **Method** | **DSC**↑ | **Recall**↑ | **Precision**↑ | **ASSD**↓ |
| --- | --- | --- | --- | --- |
| UNet_w/o_Data_preprocess | 0.820 | 0.826 | 0.820 | 1.195 |
| UNet_Data_preprocess | 0.837 | 0.844 | 0.837 | 0.613 |
| DR-LCT-UNet_w/o_Data_preprocess | 0.841 | 0.848 | 0.840 | 0.597 |
| DR-LCT-UNet_Data_preprocess | 0.858 | 0.863 | 0.858 | 0.425 |
