# Supplementary material for: Automatic coronary artery segmentation of CCTA images using UNet with a local contextual transformer
Source: Front Physiol. 2023 Aug 22;14:1138257. doi: 10.3389/fphys.2023.1138257 (PMC10478234; doi:10.3389/fphys.2023.1138257)
Supplement: Supplementary file 2 [file DataSheet1.PDF]

Supplementary materials: All of the methods that compared against in Table 3.

Case	GT	3D-UNet	VNet	ResUNet	DenseUNet	AttUNet	UNETR	UCTransNet	DR-LCT-UNet (Ours)
1	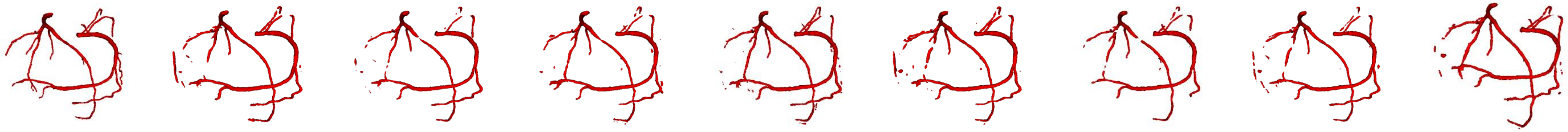								
2	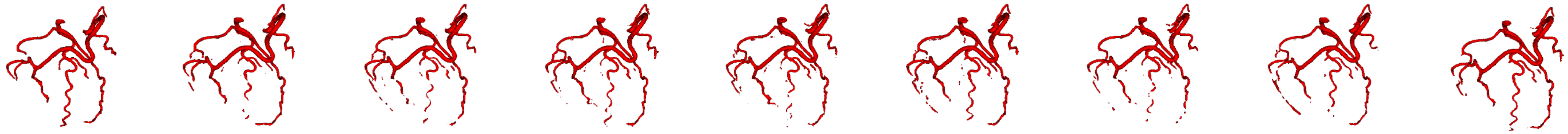								
3	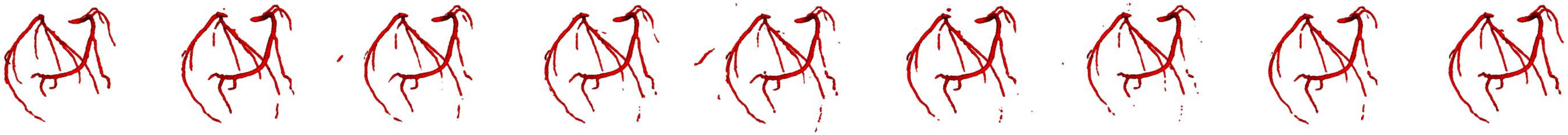								
4	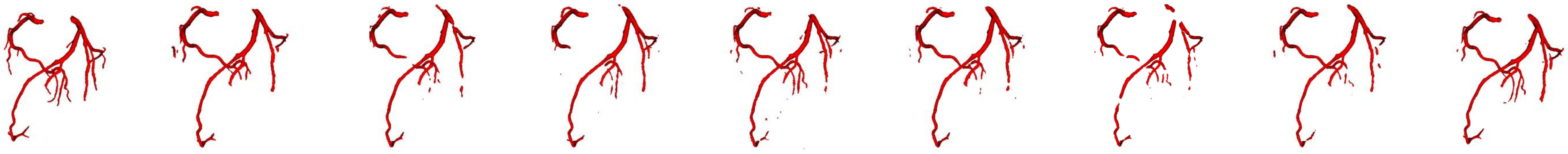								
