# Supplementary figures and images for: Automatic coronary artery segmentation of CCTA images using UNet with a local contextual transformer
Source: Front Physiol. 2023 Aug 22;14:1138257. doi: 10.3389/fphys.2023.1138257 (PMC10478234; doi:10.3389/fphys.2023.1138257)

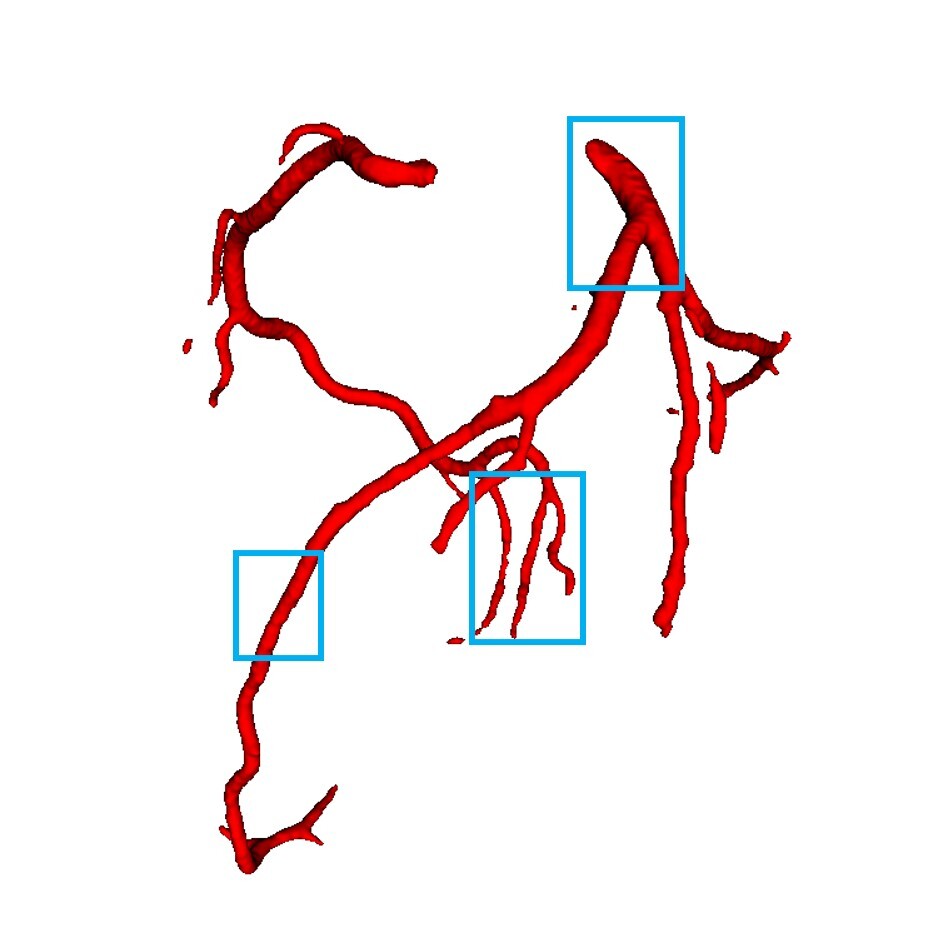

Supplement: Supplementary file 3 [file DataSheet2.zip › Supplementary Data Sheet 2/Supplementary Image 1_Segmentation result of the DR-LCT-UNet (ours) method on CCTA data of case 4.JPEG]

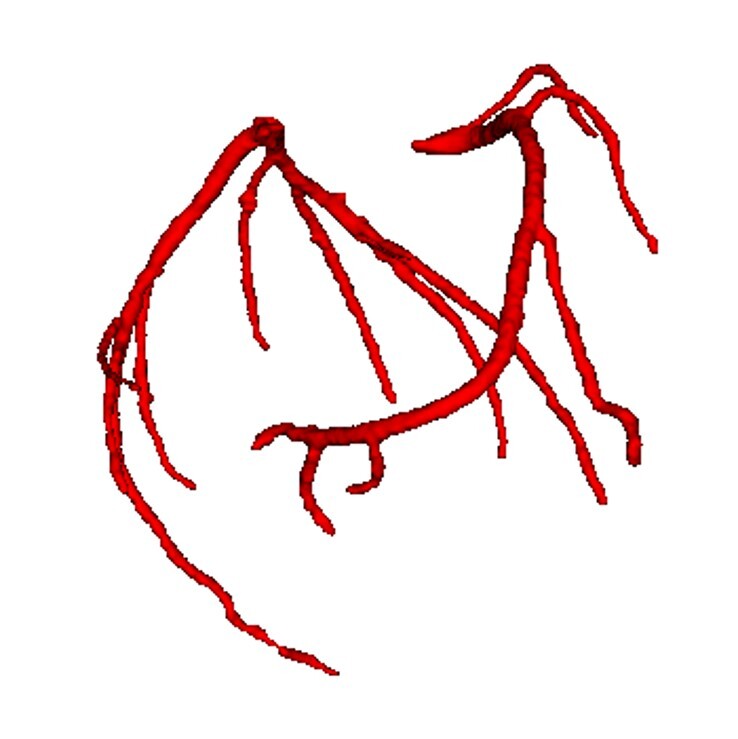

Supplement: Supplementary file 3 [file DataSheet2.zip › Supplementary Data Sheet 2/Supplementary Image 10_Ground Truth segmentation result on CCTA data of case 3..JPEG]

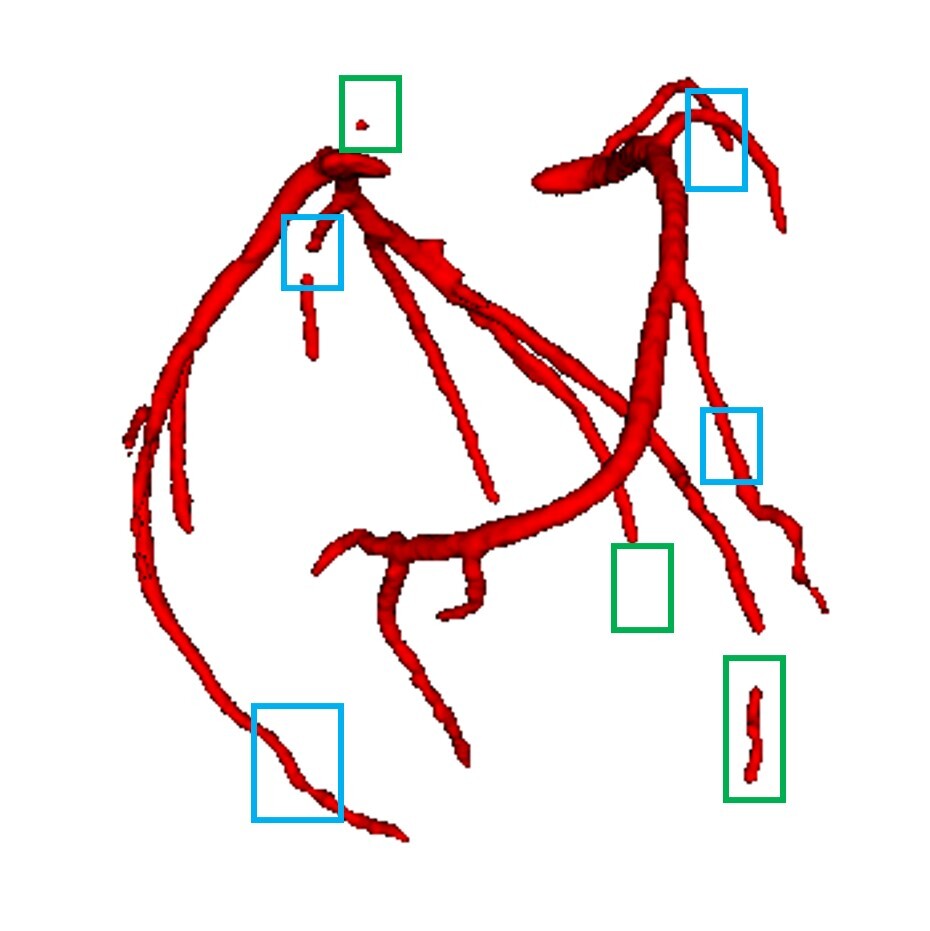

Supplement: Supplementary file 3 [file DataSheet2.zip › Supplementary Data Sheet 2/Supplementary Image 11_Segmentation result of the 3D-UNet method on CCTA data of case 3..JPEG]

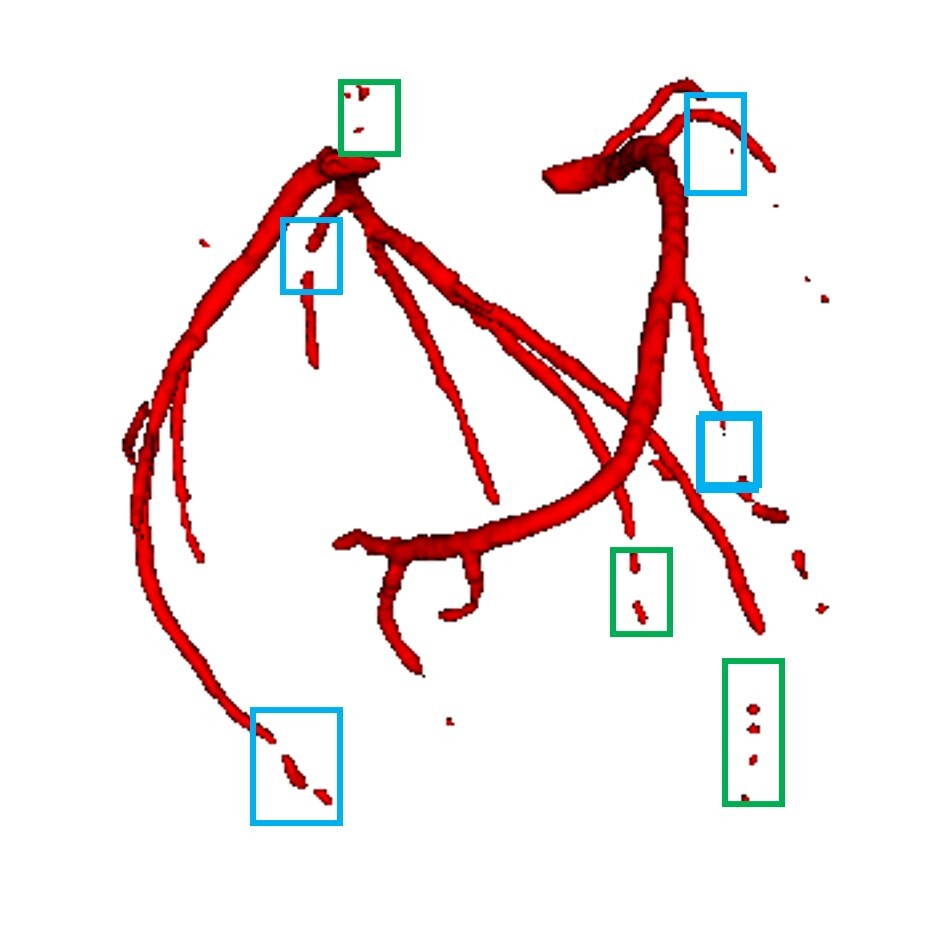

Supplement: Supplementary file 3 [file DataSheet2.zip › Supplementary Data Sheet 2/Supplementary Image 12_Segmentation result of the UNETR method on CCTA data of case 3..JPEG]

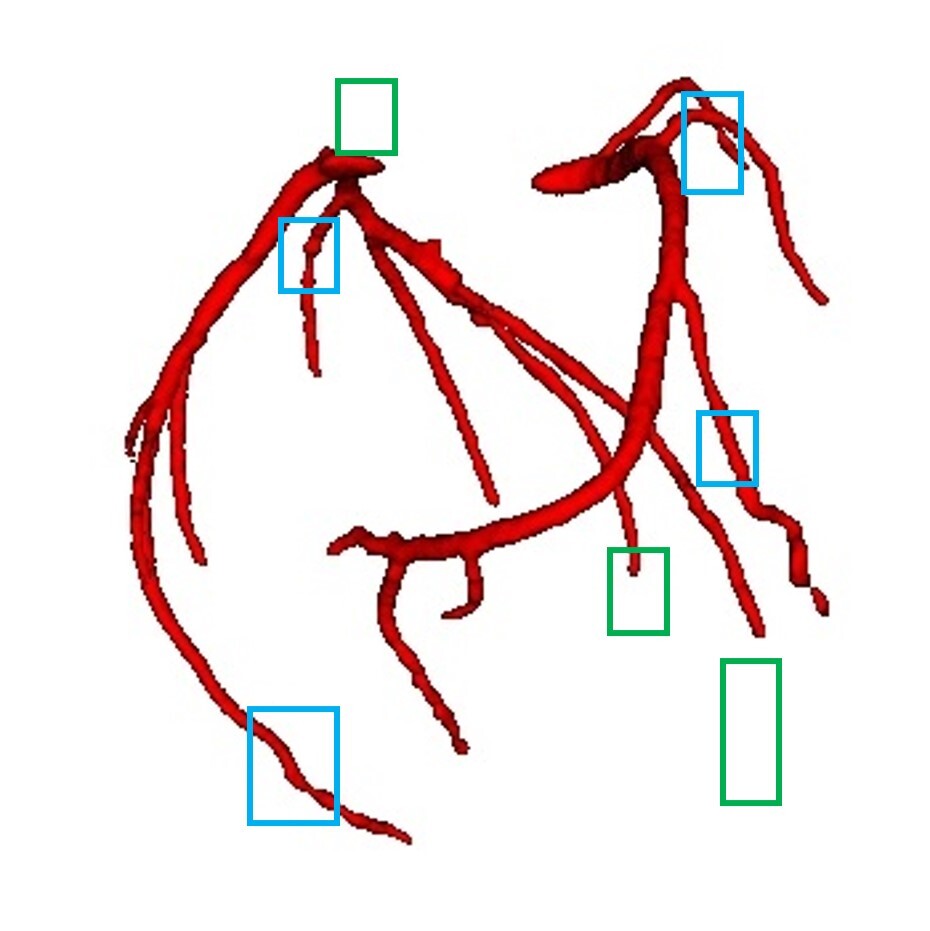

Supplement: Supplementary file 3 [file DataSheet2.zip › Supplementary Data Sheet 2/Supplementary Image 13_Segmentation result of the DR-LCT-UNet (ours) method on CCTA data of case 3..JPEG]

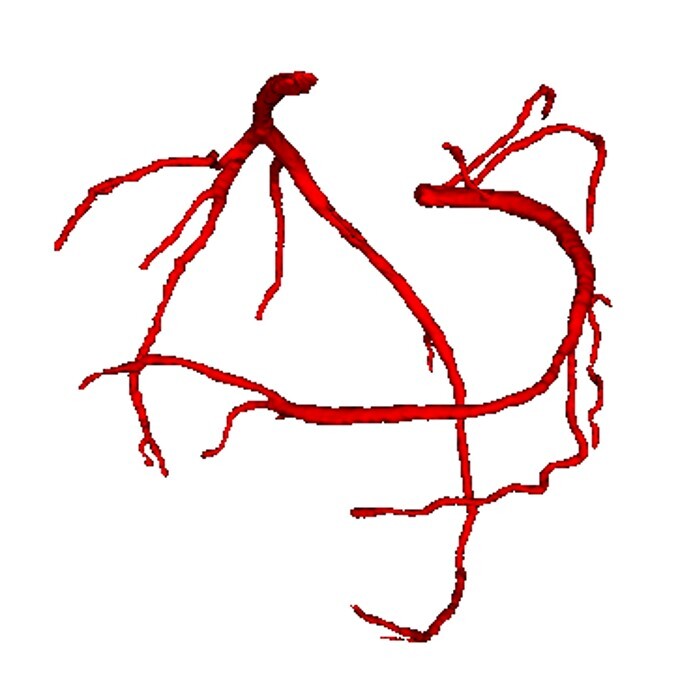

Supplement: Supplementary file 3 [file DataSheet2.zip › Supplementary Data Sheet 2/Supplementary Image 14_Ground Truth segmentation result on CCTA data of case 1..jpeg]

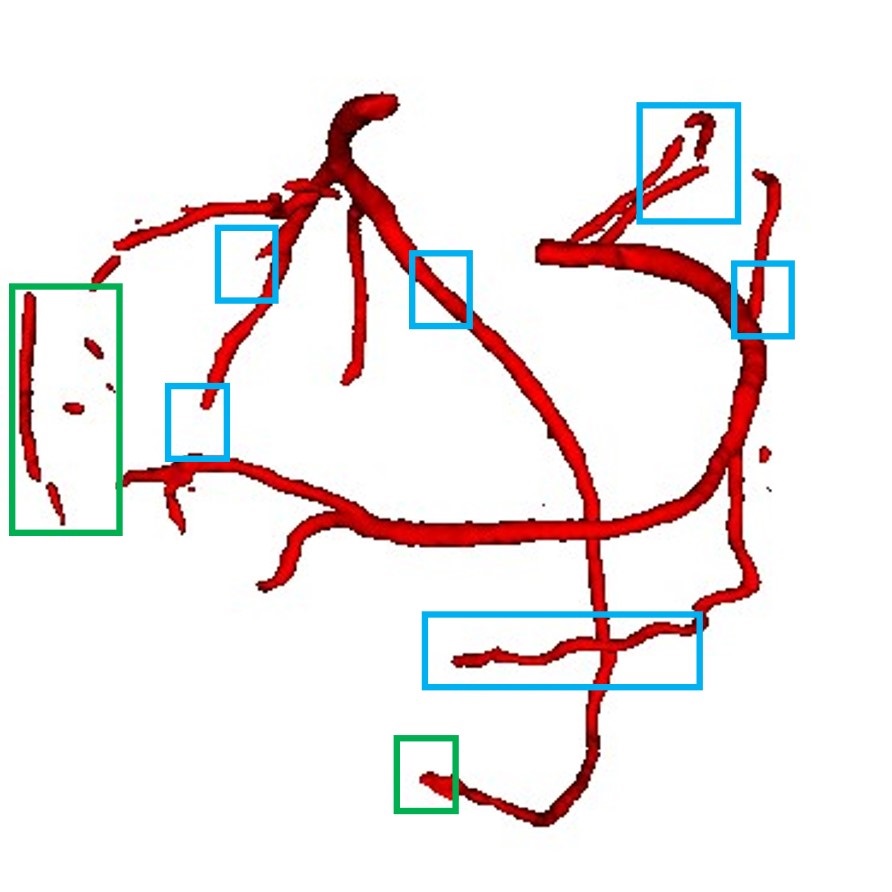

Supplement: Supplementary file 3 [file DataSheet2.zip › Supplementary Data Sheet 2/Supplementary Image 15_ Segmentation result of the 3D-UNet method on CCTA data of case 1.jpeg]

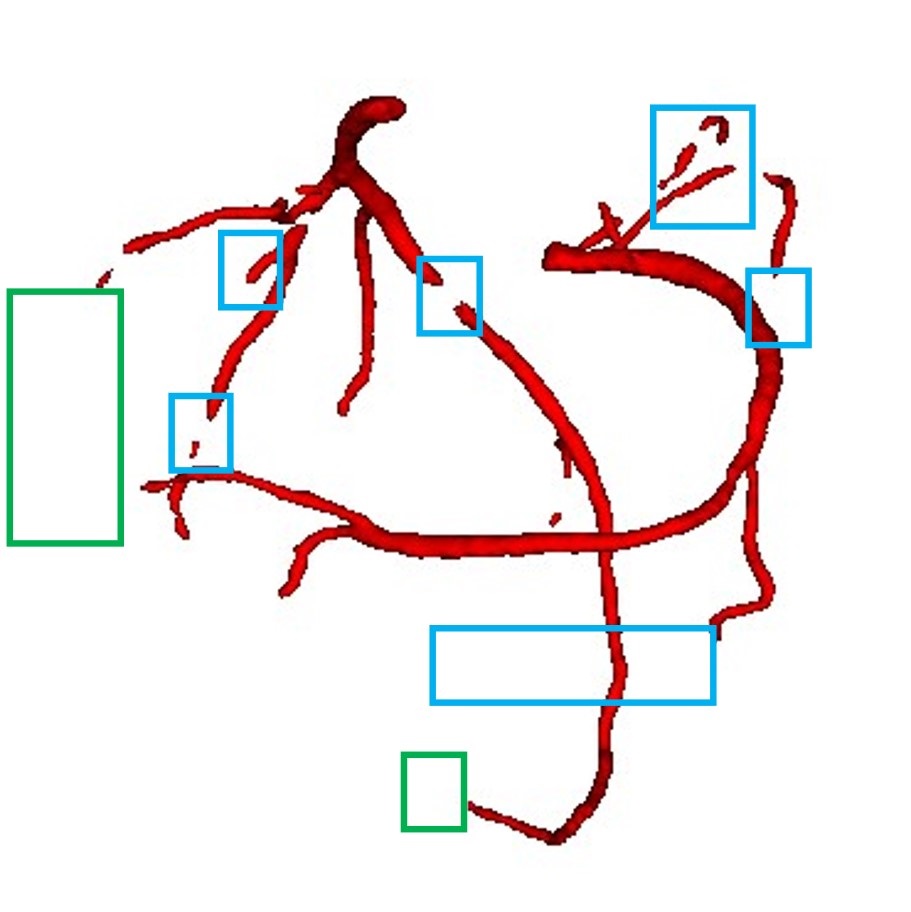

Supplement: Supplementary file 3 [file DataSheet2.zip › Supplementary Data Sheet 2/Supplementary Image 16_Segmentation result of the UNETR method on CCTA data of case 1..jpeg]

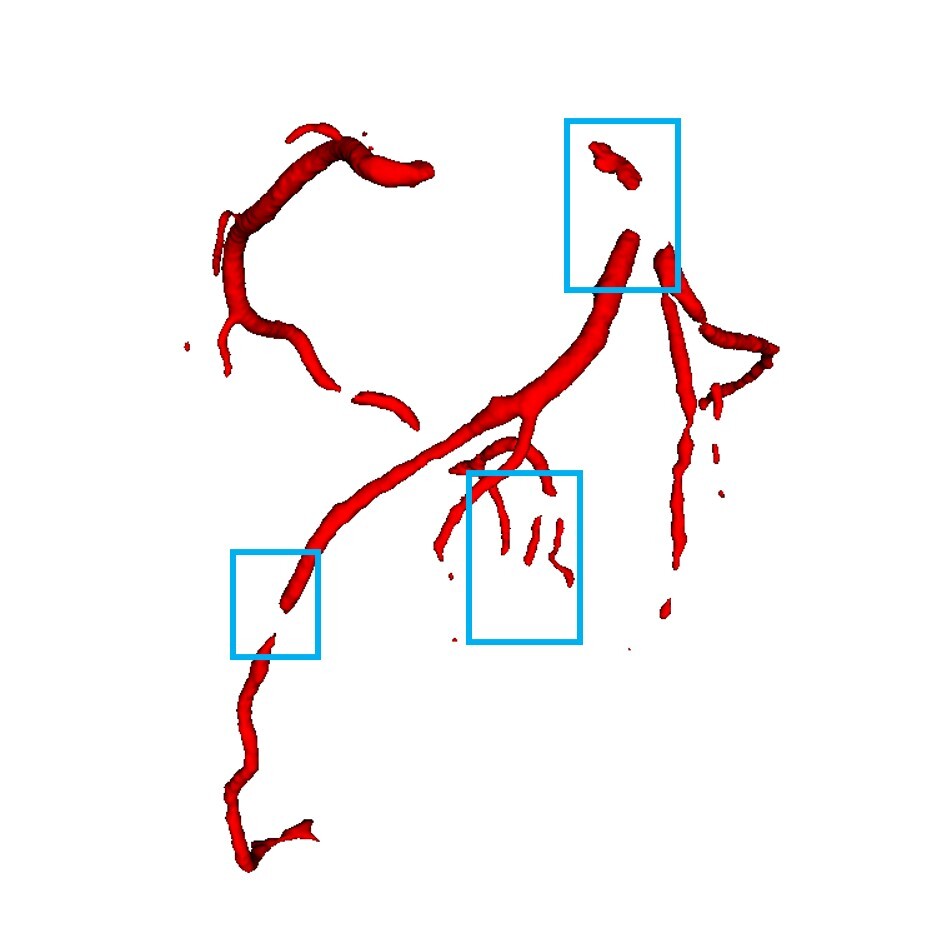

Supplement: Supplementary file 3 [file DataSheet2.zip › Supplementary Data Sheet 2/Supplementary Image 2_Segmentation result of the UNETR method on CCTA data of case 4..JPEG]

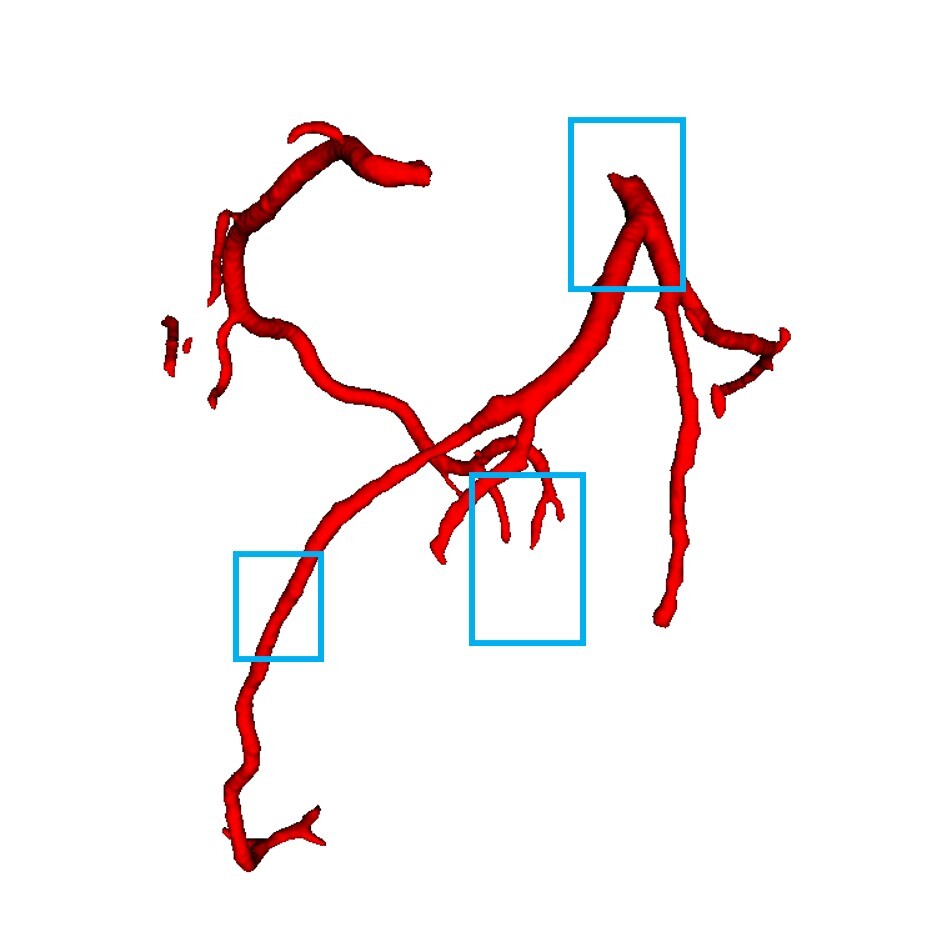

Supplement: Supplementary file 3 [file DataSheet2.zip › Supplementary Data Sheet 2/Supplementary Image 3_ Segmentation result of the 3D-UNet method on CCTA data of case 4..JPEG]

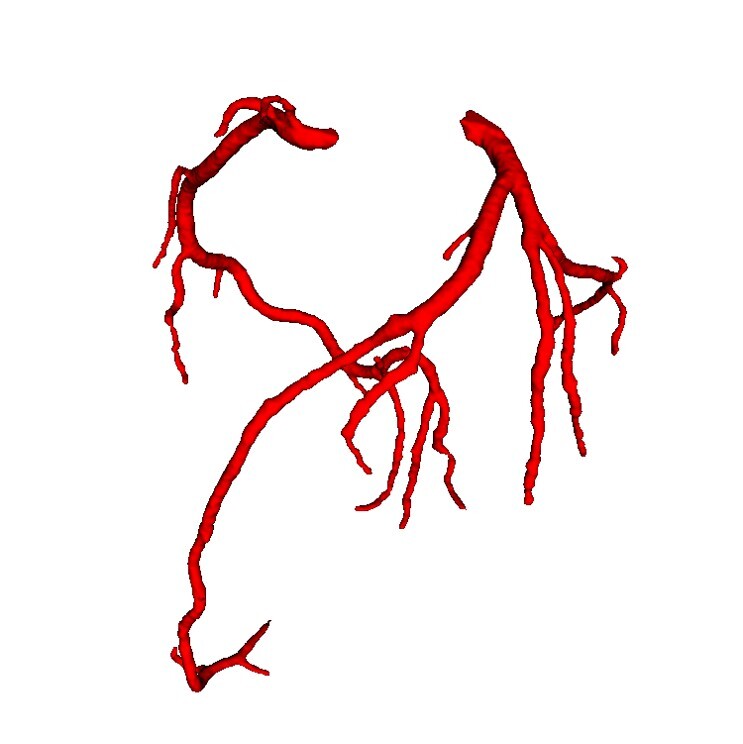

Supplement: Supplementary file 3 [file DataSheet2.zip › Supplementary Data Sheet 2/Supplementary Image 4_ Ground Truth segmentation result on CCTA data of case 4..JPEG]

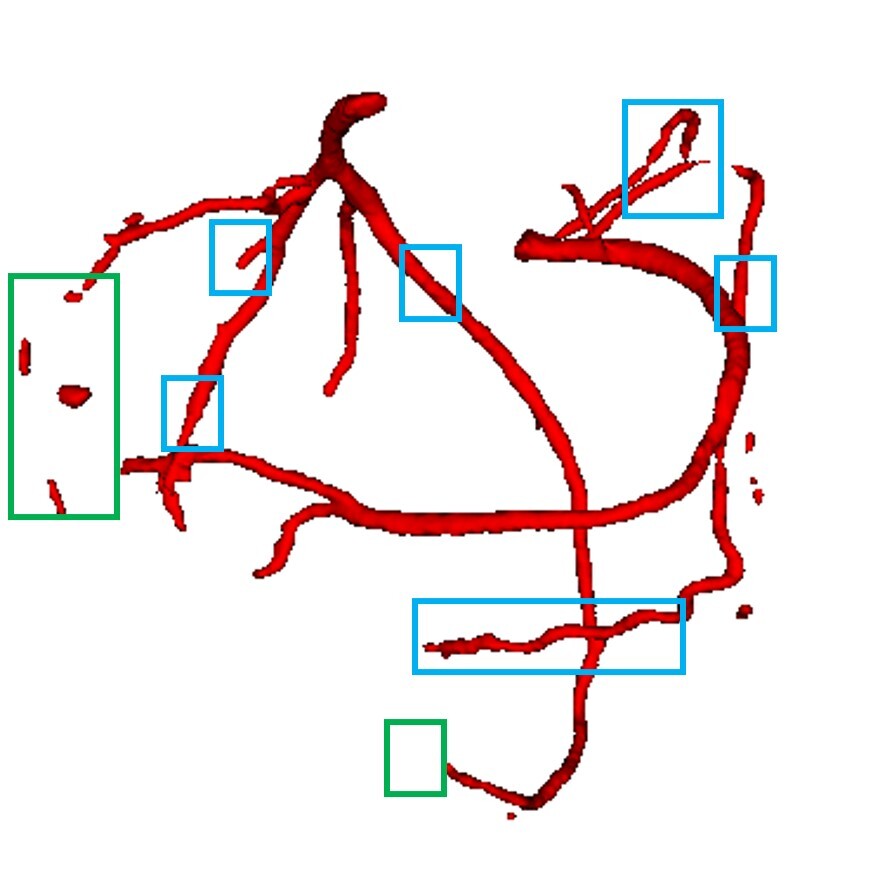

Supplement: Supplementary file 3 [file DataSheet2.zip › Supplementary Data Sheet 2/Supplementary Image 5_Segmentation result of the DR-LCT-UNet (ours) method on CCTA data of case 1..JPEG]

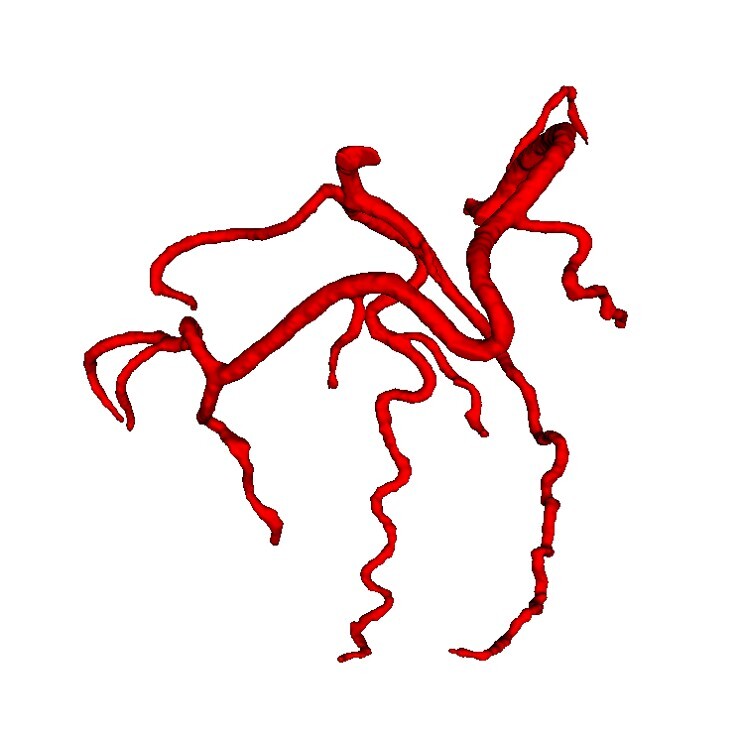

Supplement: Supplementary file 3 [file DataSheet2.zip › Supplementary Data Sheet 2/Supplementary Image 6_Ground Truth segmentation result on CCTA data of case 2..JPEG]

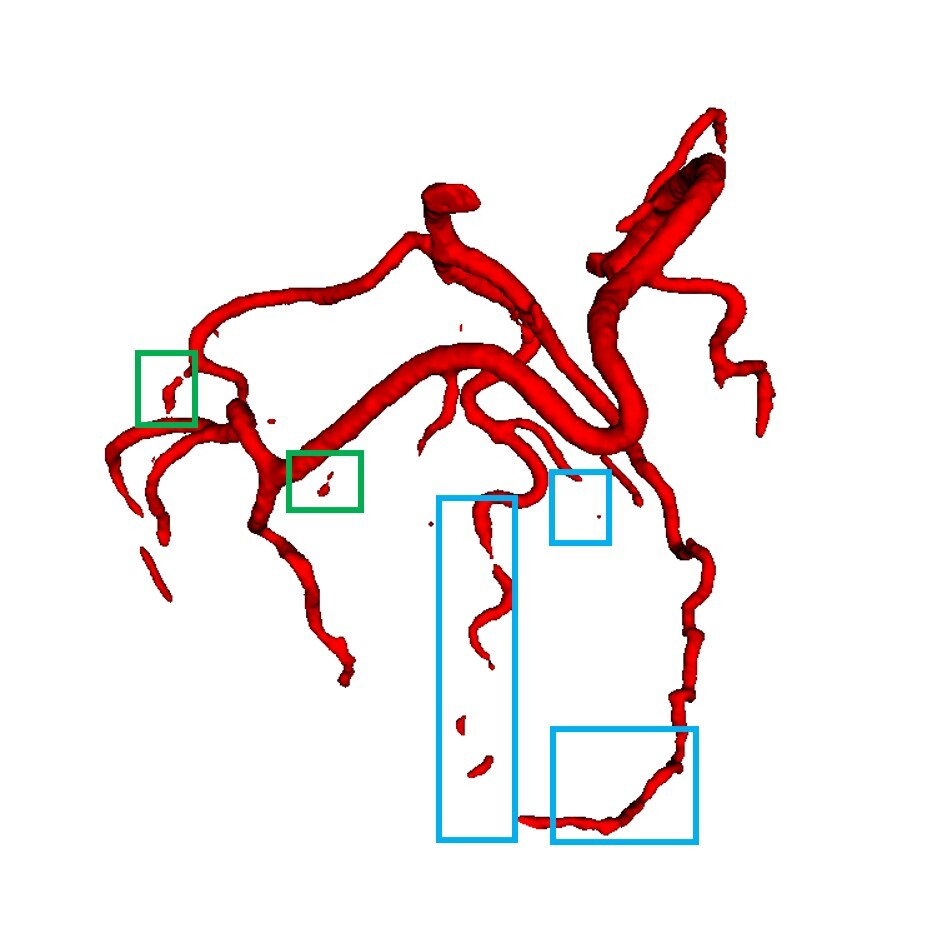

Supplement: Supplementary file 3 [file DataSheet2.zip › Supplementary Data Sheet 2/Supplementary Image 7_Segmentation result of the 3D-UNet method on CCTA data of case 2..JPEG]

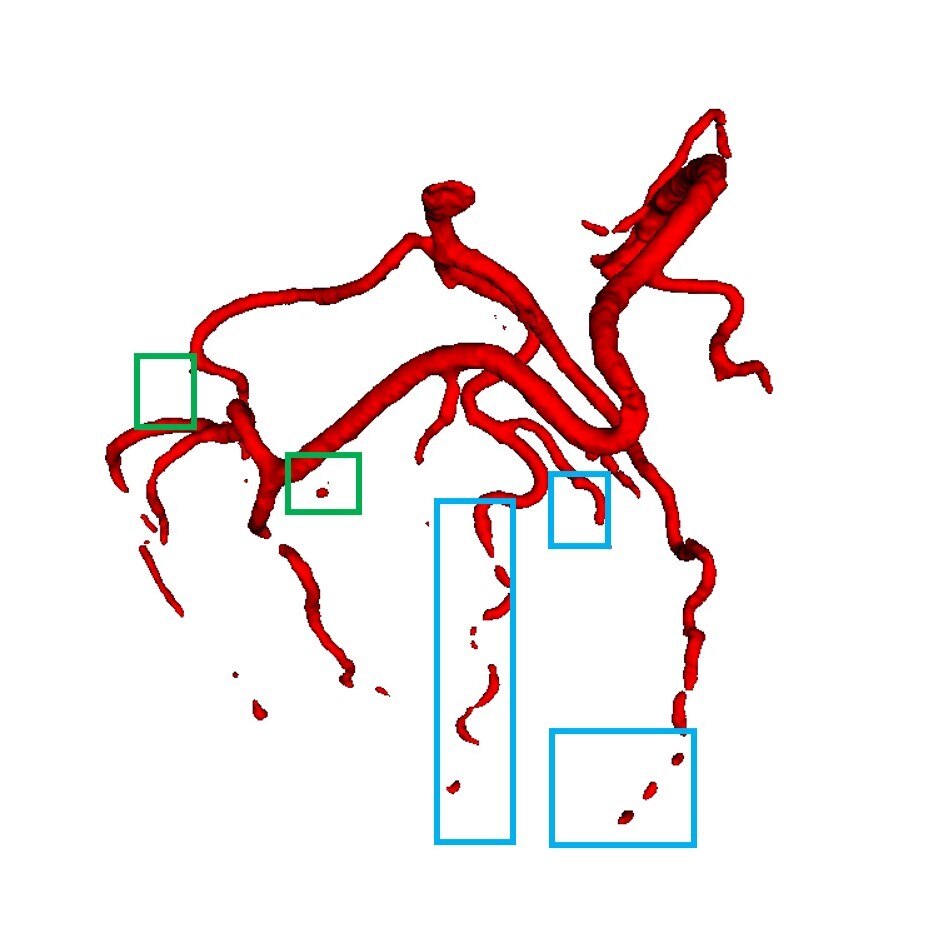

Supplement: Supplementary file 3 [file DataSheet2.zip › Supplementary Data Sheet 2/Supplementary Image 8_Segmentation result of the UNETR method on CCTA data of case 2..JPEG]

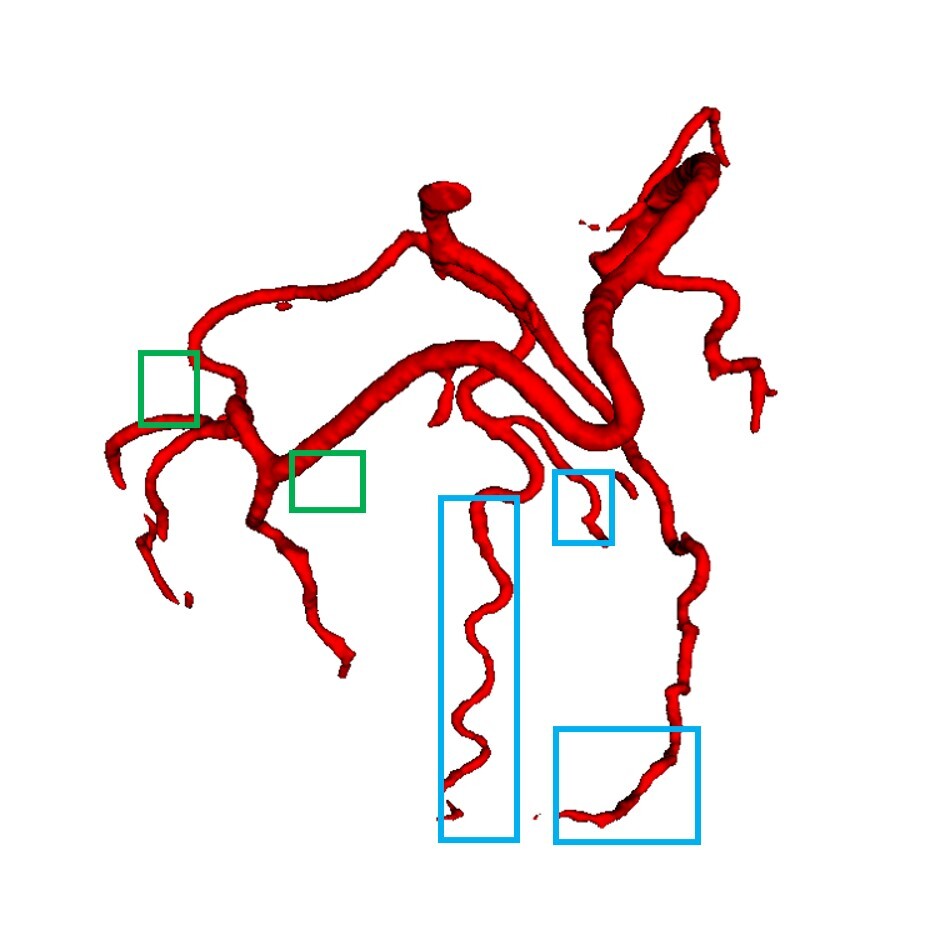

Supplement: Supplementary file 3 [file DataSheet2.zip › Supplementary Data Sheet 2/Supplementary Image 9_Segmentation result of the DR-LCT-UNet (ours) method on CCTA data of case 2..JPEG]
